# Presence and function of Hbl B’, the fourth protein component encoded by the *hbl* operon in *Bacillus cereus*

**DOI:** 10.1080/21505594.2022.2046951

**Published:** 2022-03-15

**Authors:** Nadja Jessberger, Richard Diedrich, Robert Janowski, Dierk Niessing, Erwin Märtlbauer

**Affiliations:** a Institute for Food Quality and Food Safety, University of Veterinary Medicine Hannover, Hannover, Germany; b Department of Veterinary Sciences, Faculty of Veterinary Medicine, Ludwig-Maximilians-Universität München, Oberschleißheim, Germany; c Institute of Structural Biology, Helmholtz Zentrum München-German Research Center for Environmental Health, Neuherberg, Germany; d Institute of Pharmaceutical Biotechnology, Ulm University, Ulm, Germany

**Keywords:** *Bacillus cereus*, haemolysin BL, Hbl B’, *hblB*, pore formation, cytotoxicity, enterotoxin, pore forming toxin, tripartite

## Abstract

The genes *hblC*, *hblD* and *hblA* encode the components Hbl L2, L1 and B of the pore forming enterotoxin haemolysin BL of *Bacillus cereus*. Two variants of the operon existand the more common one additionally contains *hblB* downstream of *hblCDA*. Up to now, it was completely unclear whether the corresponding protein, Hbl B’, is widely expressed among *B. cereus* strains and if it has a distinct function. In the present study, it was shown that the *hblB* gene is indeed expressed and the Hbl B’ protein is secreted by nearly all analysed *B. cereus* strains. For the latter, a detection system was developed based on monoclonal antibody 11A5. Further, a distinct reduction of cytotoxic and haemolytic activity was observed when recombinant (r)Hbl B’ was applied simultaneously with L2, L1 and B. This effect was due to direct interaction of rHbl B’ with L1. _D_^−6^*B. cereus*Altogether, we present the first simple tool for the detection of Hbl B’ in *B. cereus* culture supernatants. Moreover, an important regulatory function of Hbl B’ in the mechanism of Hbl was determined, which is best described as an additional control of complex formation, balancing the amounts of Hbl B-L1 complexes and the corresponding free subunits.

## Introduction

Hemolysin BL is a primary virulence factor produced by members of the *Bacillus cereus* group. It is involved in endophthalmitis [1,2] and causes fluid accumulation in ligated rabbit ileal loops with an activity comparable to cholera toxin [3]. Furthermore, it is dermonecrotic [4], hemolytic toward erythrocytes from several species [4–6], active in vascular permeability tests [4] and it shows cytotoxicity to a variety of different cell lines [3,7–9]. Hbl was first identified as a binary bacterial toxin consisting of a binding (B) and a lytic (L) component [5], then the three components L2, L1, and B were described [10]. In these early studies, the characteristic ring-shaped discontinuous hemolysis pattern on blood agar plates was discovered. Hemolysis occurs at an appropriate concentration ratio of Hbl L2, L1, and B [3,10,11]. Newer studies using purified recombinant (r)Hbl components and *B. cereus* culture supernatants showed that hemolysis depends on concentration and diffusion velocity of Hbl B. While rHbl L2 does not seem to have an impact on the outer hemolysis ring, it is enhanced by additional rHbl B and hindered or disrupted by additional rHbl L1 [12].

Hbl is a pore forming toxin, which was first shown by osmotic protection assays [11]. The Hbl components are structurally very similar to hemolysin E (HlyE; ClyA) from *E. coli* [13–16], which forms pores in target cell membranes by homo-oligomerization [17–19]. Nonetheless, all three Hbl components are necessary for maximum biological activity [3] and thus, to form the pore. According to the current model, the three components bind to the target cell surface sequentially in the binding order B-L1-L2 [9,12]. A defined concentration ratio is also required with most efficient pore formation at a ratio of Hbl L2:L1:B = 1:1:10 or 10:1:10 [12]. Next to the sequential binding, complex formation in solution between Hbl B monomers, Hbl B and L1, as well as Hbl L2 and L1, is also essential for rapid pore formation. It has been shown that complexation with Hbl L1 increases binding of Hbl B to the target cell surface [12,20,21]. The Hbl pore has recently been shown to be moderately cation selective and rather unstable [20]. The specific Hbl target structures LITAF and CDIP1 have also just recently been discovered on the cell surface [22].

**Figure 1.**
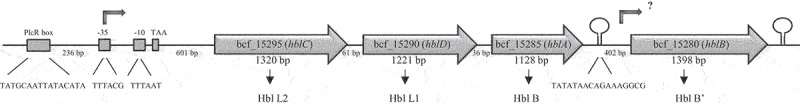
The *hbl* operon of *B. cereus* strain F837/76. The operon consists of the genes *hblC*, *D*, *A* and *B*, encoding the proteins Hbl L2, L1, B and B,’ respectively [28,32]. *hblC* and *D* are separated by 61, *hblD* and A by 36 bp. Between *hblA* and *B* is a space of 402 bp. Upstream and downstream of *hblB*, a stem loop has been detected [9]. The promoter of the *hblCDA* operon is shown, including the transcription start TAA [33]. 236 bp upstream of the promoter, a binding box of the global regulator protein PlcR has been identified [34]. The figure was adapted from Dietrich et al [24].

The *hbl* operon encoding the toxin components is found in approximately 40–70% of all known enteropathogenic *B. cereus* strains [23–28]. There are two alternatives to the *hbl* operon. The wider spread one consists of the genes *hblC* (encoding Hbl L2), *hblD* (encoding Hbl L1), *hblA* (encoding Hbl B) and *hblB* (encoding Hbl B’). The rarer variant misses the *hblB* gene. Several *B. cereus* strains harbor both variants and thus, express homologous sets of Hbl proteins [23,29–31]. In *B. cereus* strain F837/76 only the *hblCDAB* operon exists [24,32], which is shown in Figure 1. 601 bp upstream of *hblC* is the transcription start [33]. The PlcR binding box is also depicted [34]. Next to the global phospholipase C regulator, expression of the *hbl* operon is further regulated, probably in a concerted action, by CcpA, CodY, SinR, ResD and Fnr [24]. The genes *hblC*, *D*, *A,* and *B* have a size of 1320, 1221, 1128, and 1398 bp. Between *hblC* and *D* is a space of 61 nucleotides, between *hblD* and *A* 36, while the genes *hblA* and *B* are separated by 402 nucleotides. Upstream and downstream of *hblB,* a stem loop has been identified, which could function as transcriptional terminator [9]. Furthermore, a PlcR-independent transcription of *hblB* has been suggested [35]. *hblB* was first found by sequencing the *hblA* gene of *B. cereus* strain F837/76 [36]. As the C-terminal part of *hblB* and *hblA* differs significantly, it was stated that *hblB* emerged from duplication of *hblA* and a C-terminal fusion with another ORF [34].

Transcription of *hblCD*A was found to end within [33,37,38] or upstream [9] of *hblB*, until Clair et al. demonstrated that the gene is transcribed, translated, and the protein exported [35]. The study also suggested that Hbl B’ accumulates in *B. cereus* supernatants in lower amounts than the other Hbl components, which might be a reason that it has not been detected earlier. Furthermore, a 2-fold upregulation of *hblB* in hyperflagellated swarm cells of *B. cereus* strain ATCC 14579 was found [39]. Although it has now been shown that Hbl B’ is indeed produced and secreted by *B. cereus*, it is still unclear if it has a certain function. Due to the sequence homology of Hbl B, it has been speculated that Hbl B’ could complement or reinforce the function of Hbl B under specific growth conditions [35].

Just recently, we were able to overexpress and purify fully functional recombinant Hbl components (rHbl) from *E. coli* and to study the mode of action of Hbl in more detail [12,20,21]. The aim of the current study was (i) to develop a system for the detection of Hbl B’ in *B. cereus* culture supernatants and (ii) to investigate its putative involvement in Hbl activity and thus, in virulence of *B. cereus*.

## Materials and methods

### Bacterial strains and culture conditions

The following, previously well-characterized *B. cereus* strains were used in this study: F837/76, F837/76 ∆*nheABC*, F4430/73, 14294–3 (M6), SDA KA 96, INRA A3, INRA C3, 6/27/S, F3175/03 (D7), RIVM BC 934, F528/94, RIVM BC 126, NVH 0075–95, and MHI 226 [21,23,40–43]. For RNA preparation, the strains were cultivated for 3 h in CGY (casein-glucose-yeast) medium with 1% glucose before centrifugation (15 min, 4°C 4000 g) and disposal of the supernatant. For a collection of toxin-rich supernatants, the strains were grown for 6 h in CGY medium. After centrifugation, 1 mM EDTA was added, and the supernatants were filtered through 0.2 mm sterile filters. For the overexpression of the rHbl components L2, L1, B and B,’ the *E. coli* strain BL21 (DE3) transformed with the corresponding pASK-IBA plasmids was grown in LB medium with 100 µg/ml ampicillin.

### Cell lines and culture conditions

Vero cells were purchased from ECACC (European Collection of Cell Cultures), CaCo-2 and A549 cells from DSMZ (German Collection of Microorganisms and Cell Cultures, Braunschweig, Germany). They were maintained in 80 cm^2^ culture flasks in a humidified incubator at 37°C and 7% CO_2_ as recommended by the supplier.

### Preparation of total RNA

Total RNA preparation and on-column DNase digestion were performed using peqGOLD Total RNA and peqGOLD DNase I Digest Kit (peqlab, Erlangen, Germany) according to the instructions of the manufacturer. Electrophoresis on agarose gels and testing in a spectrophotometer revealed the quality of the RNA. RNA purity was confirmed via PCR for a 241 bp 16S rRNA fragment using the primers 16S_fw (GGAGGAAGGTGGGGATGACG) and 16S_rev (ATGGTGTGACGGGCGGTGTG).

### Reverse transcription and *hblB* PCR

Using 100 ng RNA as template, double-stranded cDNA was synthesized with the ProtoScript first-strand cDNA synthesis kit (New England Biolabs, Frankfurt, Germany) and random primer mix according to the instructions of the manufacturer. The cDNA was subsequently applied to a PCR for a 166 bp fragment of *hblB* using the primers hblB-3'-fw (CATAACG CATACACTTTTGAAATAAAG) and hblB-3'-rev (CCGCAAATTCATCATTTGGATTG). The fragment represents the 3' part of the gene not identical to *hblA.*

### Cloning, overexpression, and purification of recombinant Hbl B’

Recombinant Hbl L2, L1, and B were produced as described before [21]. The *hblB* gene, encoding the putative fourth component of the *B. cereus* Hbl toxin, was amplified from chromosomal DNA of strain F837/76 using the primers HblB’-fw-KpnI (ATATGGTACCCGCAATTGAAATTCAACAAACG) and HblB’-rev-NcoI (ATATCCATGGTCAGTTCAT TATATTTTGTACTTTG). The amplified gene lacked the sequence for the N-terminal signal peptide for secretion [34]. It was cloned into the vector pASK-IBA5plus (IBA Lifesciences, Göttingen, Germany), which added an N-terminal strep-tag to the recombinant Hbl B’ protein. Analogously to that, a truncated rHbl B’ protein was produced lacking the C-terminal 91 amino acids. For that, the same forward primer as well as HblB’(tr)-rev-NcoI (ATATCCATGGTCAATTCAAAAAATCCTGTTCTTTTTTCTC) as reverse primer was used. Similarly to the full-length *hblB* of F837/76, the *hblB* genes of the other 10 *B. cereus* strains (see above) were amplified and cloned. Overexpression and purification of the rHbl B’ proteins were performed analogously to Hbl L2, L1, and B as described before [21].

### SDS PAGE and Sypro staining

SDS-PAGE was run on a PhastGel gradient (10–15%) minigel system (GE Healthcare, Munich, Germany). For Sypro staining, proteins were fixed on the gel for 2 × 30 min in 50% MeOH and 7% acetic acid. The gel was then incubated with 2 ml Sypro Ruby protein stain (Sigma-Aldrich; Merck, Darmstadt, Germany) overnight at room temperature. After 30 min washing in 10% MeOH and 7% acetic acid and additional washing for 10 min in H_2_O, fluorescence signals were detected on a UVP ChemStudio imager (Analytik Jena, Jena, Germany).

### Western blotting

For Western blotting, 30 µl protein solution or *B. cereus* culture supernatant were applied to Criterion XT precast gels (BioRad Laboratories, Feldkirchen, Germany). After electrophoresis, proteins were blotted to a PVDF-P membrane (Millipore; Merck, Darmstadt, Germany). The membrane was saturated with 3% casein-PBS and incubated for 1 h at room temperature with 2 µg/ml Strep-MAB-Classic (IBA Lifesciences, Göttingen, Germany) or monoclonal antibody (mAb) 11A5 [21] for detection of Hbl B.’ Afterward, it was washed three times in PBS with 0.1% Tween 20 and incubated overnight with a 1:2000 dilution of rabbit anti-mouse-horseradish peroxidase conjugate (Dako; Merck, Darmstadt, Germany) before three further washing steps in PBS with 0.1% Tween 20 and two in PBS. Subsequently, Super Signal Western Femto (Pierce; Thermo Fisher Scientific, Waltham, MA, USA) was applied, and chemiluminescence signals were sensed on a UVP ChemStudio imager (Analytik Jena, Jena, Germany).

### Cytotoxicity assays

1.5 pmol/µl stock solutions of the rHbl components L2, L1, B, B’, and B’(tr) were used in toxicity assays. If not stated otherwise, they were added in 1:40 dilutions or as dilution series to the cells after pre-mixing in appropriate ratios. WST-1 bioassays and propidium iodide (PI) influx tests were performed as previously described [7,12,21,42,46,47].

### Determination of hemolytic activity

Ten µl of *B. cereus* supernatants or rHbl components (1.5 pmol/µl each) were applied as mixtures or individually into 3.5 -mm diameter stamp holes on sheep blood agar plates. If not stated otherwise, they were photographed after 3–6 h of incubation at 32°C. For quantification, samples were applied to hemolysis assays with defibrinated sheep blood (Oxoid; Thermo Fisher Scientific, Waltham, MA, USA). The blood was applied to centrifugation (5 min at RT and 590 g) and washed three times in 150 mM NaCl, 5 mM Tris-HCl, pH 7.2. After that, it was diluted to 2% in the buffer. Two µl *B. cereus* supernatant or 10 µl rHbl samples were added to 800 µl total volume. As negative and positive controls, erythrocytes in 800 µl buffer and in 800 µl H_2_O were carried along, respectively. Samples (triplicates) were incubated for 20 min (supernatant) or 30 min (rHbl) at 37°C and subsequently centrifuged for 1 min at RT and 8000 g. Subsequently, an Infinite F50 photometer (Tecan Group Ltd., Männedorf, Switzerland) was used to determine the optical density at 562 nm. Results were compared to the positive control, which was set to 100%.

### Flow cytometry

1 × 10^6^ Vero cells in 500 µl buffer (140 mM NaCl, 15 mM HEPES, 1 mM MgCl_2_, 1 mM CaCl_2_, 10 mM glucose, pH 7.2) per sample were used. rHbl B, rHbl B’, and rHbl B’(tr) were applied at a concentration of 9.38 pmol/ml in the cell culture medium to a total volume of 1 ml and incubated for 1 h at 37°C under gentle agitation. Similarly, 1:1 mixtures of rHbl B and L1, L2, or B’ were used. After two washing steps in 2 ml 1% BSA-PBS and centrifugation for 5 min at 800 rpm, 5 µg/ml mAb 11A5 was added for 1 h at RT. Samples were again washed twice and subsequently incubated with 2 µg/ml Alexa Fluor® 488 goat antimouse IgG (Life Technologies; Thermo Fisher Scientific, Waltham, MA, USA) for 45 min at 4°C. Alternatively, directly labeled mAbs 1B8- or 1G8-Alexa Fluor® 488 were used. Two additional washing steps were carried out. After that, cell pellets were resuspended in 500 µl 1% BSA-PBS and transferred to flow cytometry tubes. FACS Calibur and CellQuestPro software (BD Bioscience, Heidelberg, Germany) were used for fluorescence measurements. Results were analyzed using Flowing Software (Turku Bioscience, https://bioscience.fi/services/cell-imaging/flowing-software/). Cell populations were visualized in the FSC SSC (forward scatter – side scatter) dot plot. Fluorescence was recorded in FL1 (fluorescence channel 1) and is shown as histogram.

### Surface Plasmon Resonance (SPR)

Measurements were performed on a Biacore S200 instrument (Cytiva Europe GmbH, Freiburg, Germany). rHbl B’ was diluted to a final concentration of 150 nM in 10 mM sodium acetate, pH 4.8, and chemically immobilized (amine coupling, 350 RU bound) onto series S sensor chip CM5 (Cytiva Europe GmbH, Freiburg, Germany). rHbl L1, L2, and B protein samples were diluted in a running buffer (PBS, 1 mM DTT and 0.005% Tween 20) to the final concentration of 7.8125 nM, 15.625 nM, 31.25 nM, 62.5 nM, 125 nM, 250 nM, 500 nM, 1 µM, and 2 µM and injected over the sensor chip surface at 30 µl/min at 20°C from the lowest to the highest concentration. Injection of 250 nM protein concentration was always performed in duplicate within each experiment. For the sensor chip regeneration after each injection, 1 M MgCl_2_ solution has been used for rHbl L1 and 0.5 M NaCl solution for rHbl L2 and B. In order to subtract any background noise from each experiment, all samples were also run over an unmodified sensor chip surface. Data analysis was performed with Biacore S200 evaluation software v1.1. For each measurement, the equilibrium dissociation constant (K_D_) was calculated using a 1:1 binding model. The K_D_s from three experiments were used to calculate the mean values of these variables and the SEM.

### *In silico* analyses

DNA and protein sequence alignments were generated using clustal omega [44]. Structural models of Hbl B and B’ of *B. cereus* strain F837/76 were gained using SWISS-MODEL [45].

## Results

### The *hblB* gene is expressed in 12 different *B.* *cereus* strains

**Figure 2.**
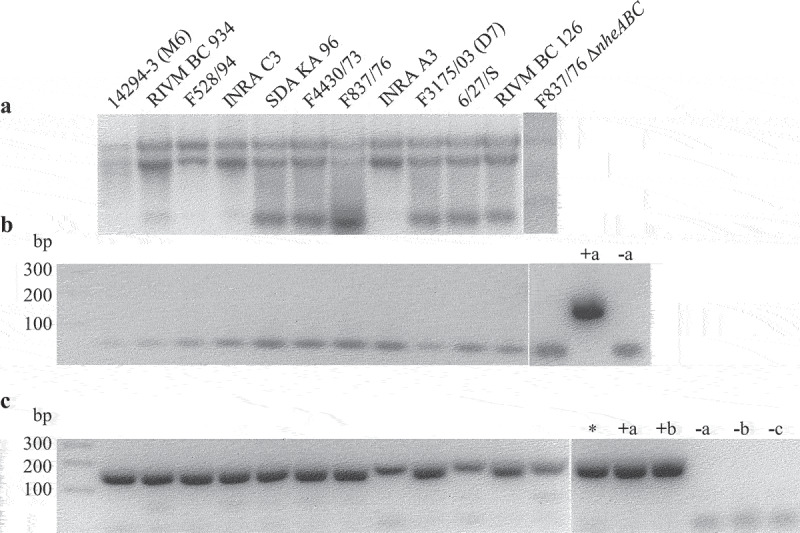
Detection of *hblB* expression in 12 different *B. cereus* strains. Strains were grown for 3 h in CGY full medium before preparation of total RNA. (a). Control of RNA integrity on 1% agarose gel. (b). Control PCR of a 241 bp fragment of the 16S rRNA gene. Only primers were visible. Negative PCR results revealed no residual DNA in the samples. Chromosomal DNA from F837/76 served as positive control (+a), H_2_O as negative control (−a). (c). PCR amplification of a 166 bp fragment of *hblB* after reverse transcription of the RNA samples. The fragment represents the 3' part of the gene not identical with *hblA* . Chromosomal DNA from F837/76 (+a) as well as INRA A3 (+b) served as positive, H_2_O as negative control (−a). Chromosomal DNA samples of strains NVH 0075–95 (Nhe reference, no *hbl* genes, named -b) and *hblB*-negative strain MHI 226 (−c) did also not result in a PCR product. *: cDNA of strain F837/76 grown under simulated intestinal conditions in a previous experiment.

Earlier, contradictory studies [28,35] raised the question if the *hblB* gene is generally transcribed and the corresponding protein expressed in *B. cereus*. To answer that, total RNA was prepared from different *B. cereus* strains with known enterotoxin gene profiles [23,40,42], which were grown for 3 h in CGY medium (Figure 2a). After a control PCR for a 16S rRNA gene fragment revealed no residual DNA (Figure 2b), RNA was reverse transcribed into cDNA and subsequently used for PCR amplification of a 166 bp fragment of *hblB*. The *hblB* fragment could be detected for all tested *B. cereus* strains (Figure 2c), indicating that *hblB* is indeed transcribed under ideal growth conditions. Moreover, the *hblB* fragment was also detected when cDNA from a previous study was used, in which strain F837/76 was grown under “simulated intestinal conditions” (RPMI 1640 medium, 37°C 7% CO_2_ atmosphere) [43]. As a positive control, chromosomal DNA of strains F837/76 and INRA A3 was used. Chromosomal DNA of Nhe reference strain NVH 0075–95 [48] as well as MHI 226 bearing only a truncated *hblCDA* operon [23] revealed the specificity of the reaction (see also Figure 2c).

### mAb 11A5 detects recombinant (r)Hbl B and rHbl B’ in Western blots

**Figure 3.**
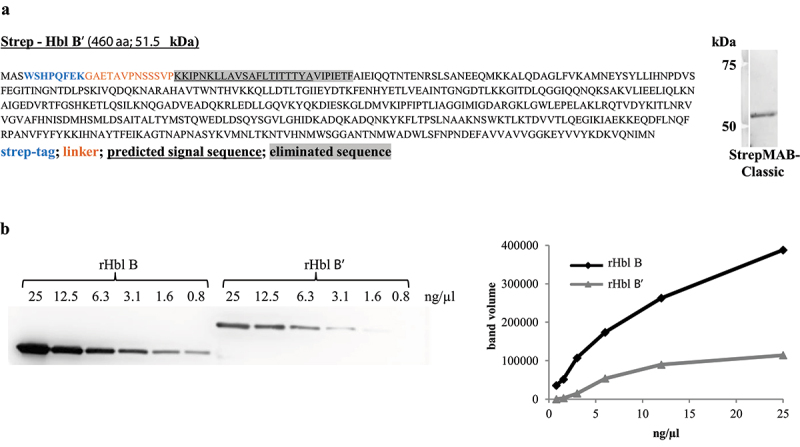
Binding of mAb 11A5 to rHbl B and B.’ (a). Purification of rHbl B’ via N-terminal strep-tag. In the amino acid sequence, the strep-tag is depicted in blue, the linker in orange letters. The predicted signal sequence for secretion [34] is underlined and eliminated amino acids are highlighted in gray. The predicted molecular weight is 51.5 kDa. The protein was detected via Western blot using the strep-tag-specific StrepMAB-Classic. (b). Western blot with decreasing amounts of the rHbl B and B’ proteins. The two proteins are simply distinguishable according to their molecular weight (rHbl B: 40.9 kDa, rHbl B:’ 51.5 kDa). Blackness values are depicted in proportion to the utilized protein concentrations.

Analogously to the three recently purified rHbl components [21], the *hblB* gene from *B. cereus* strain F837/76 was cloned into the vector pASK-IBA5plus. Thereby, the N-terminal signal peptide for secretion was dismissed, and an N-terminal strep-tag was added. *E. coli* BL21 (DE3) was used for protein overexpression. Purification was conducted via affinity chromatography. The 460 amino acid protein with a predicted molecular mass of 51.5 kDa was detected by Western blotting using an anti-strep mAb (Figure 3a). Subsequently, the generated rHbl B’ protein was used to screen a set of Hbl B-specific mAbs [21,46] for binding. Of the three potential candidates, mAb 11A5 was best suited for the specific detection of rHbl B and B’ in Western blots at 40.9 and 51.5 kDa, respectively. From the band intensities, the relative affinity of mAb 11A5 was estimated, which was approximately 3x higher for rHbl B than B’ (Figure 3).

### Hbl B’ is present in the culture supernatant of 10 *B.* *cereus* strains

**Figure 4.**
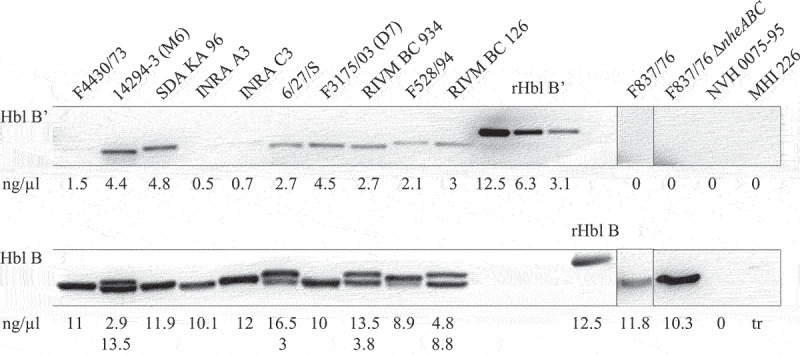
Detection of Hbl B and B’ in the supernatant of several *B. cereus* strains. Western blots were performed using specific mAb 11A5. After 20 s exposure, strong signals for Hbl B appeared. Thus, the membrane was cut and Hbl B’-specific signals were detected after an additional 3 min exposure. This procedure is possible due to the molecular weights of Hbl B (approx. 40 kDa) and Hbl B’ (approx. 50 kDa). The recombinant proteins, which were used as concentration standards, appeared slightly bigger in the gel than the native proteins, which is due to their additional strep-tag (refer to Figure 3). Shown is one representative blot per strain. Protein concentrations were calculated according to rHbl B and rHbl B’ concentration standards as mean of 2–4 individual blots. For strains F837/76 and F837/76 ∆*nheABC*, no Hbl B’ protein could be detected. NVH 0075–95 and MHI 226 were applied as negative controls. 0: no detectable signal. tr: traces.

For the detection and estimation of the amounts of Hbl B’ in *B. cereus* culture supernatants, Western blots were again carried out using mAb 11A5. Supernatants of the 12 aforementioned *B. cereus* strains were investigated, now after 6 h growth in CGY medium. Strong signals for Hbl B and weaker signals for Hbl B’ were detected according to the mAb’s relative affinity (see above). Protein concentrations were calculated according to rHbl B and rHbl B’ concentration standards (Figure 4). Hbl B was detected in the supernatants of all 12 strains with average protein levels varying between 2.9 and 16.5 ng/µl. Moreover, two distinct bands for Hbl B were detected for strains 14294–3 (M6), 6/27/S, RIVM BC 934, and RIVM BC 126, which might correspond to two different versions of the *hbl* operon partly found in their genomes. Hbl B’ was also detected in the supernatant of most strains. Protein levels varied between 0.5 and 4.8 ng/µl. Surprisingly, F837/76 and F837/76∆*nheABC* showed no signals for Hbl B.’ NVH 0075–95 (*hbl*-negative) and MHI 226 (truncated *hblCDA* operon) were again applied as negative controls.

### rHbl B’ cannot replace any of the other Hbl components

**Figure 5.**
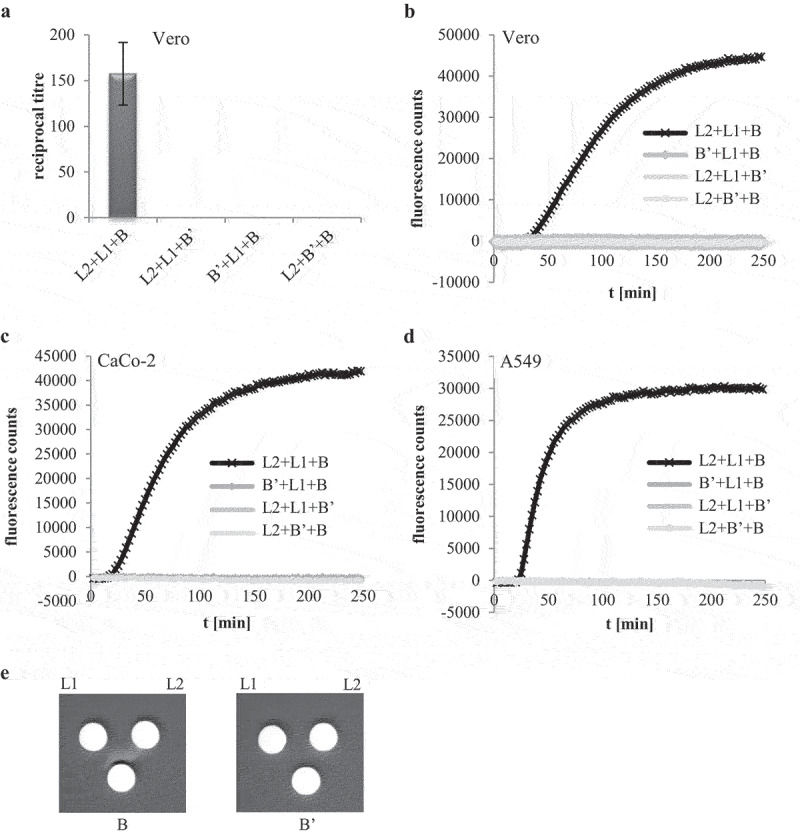
Hbl B’ does not substitute for other Hbl components. rHbl components L2, L1, B and B’ were set to 1.5 pmol/µl each and mixed in 1:1:1 ratios. (a). For WST-1 bioassays on Vero cells the pre-mixed rHbl components were applied as dilution series. (b). For measurement of PI influx into Vero cells the pre-mixed rHbl components were used in 1:40 dilution. (c). PI influx test on CaCo-2 cells. Samples were applied as in B. (d). PI influx test on A549 cells. Samples were applied as in B. (e). Ten µl of each protein component were pipetted into a stamp hole in sheep blood agar plates.

based detection system for Hbl B,’ the recombinant protein was also used in different (cell culture) assays to determine its influence on Hbl activity. Pore formation and cytotoxic activity of the rHbl components L2, L1, and B have already been shown in PI (propidium iodide) influx tests and WST-1 (water-soluble tetrazolium) bioassays on target cells, as well as on planar lipid bilayers [12,20,21]. Because of the sequence homology between Hbl B and Hbl B’ [34] , the latter was considered as a putative substitute for at least Hbl B. In Figure 5 it is shown that rHbl B’ is neither able to replace rHbl B nor rHbl L2 or L1. This observation was made in WST-1 bioassays (Figure 5), where cell viability is determined after 24 h of incubation, as well as in PI influx tests (Figure 5), where pore formation into the target cell membrane is measured. Consistent data were obtained on Vero, CaCo-2, and A549 cells (see Figure 5), indicating that the failure of rHbl B’ to substitute the other components does not depend on the target cell line. Also, no hemolytic activity was observed on sheep blood agar plates when rHbl B was substituted with rHbl B’ (Figure 5).

### rHbl B’ reduces cytotoxic and hemolytic activity of Hbl

**Figure 6.**
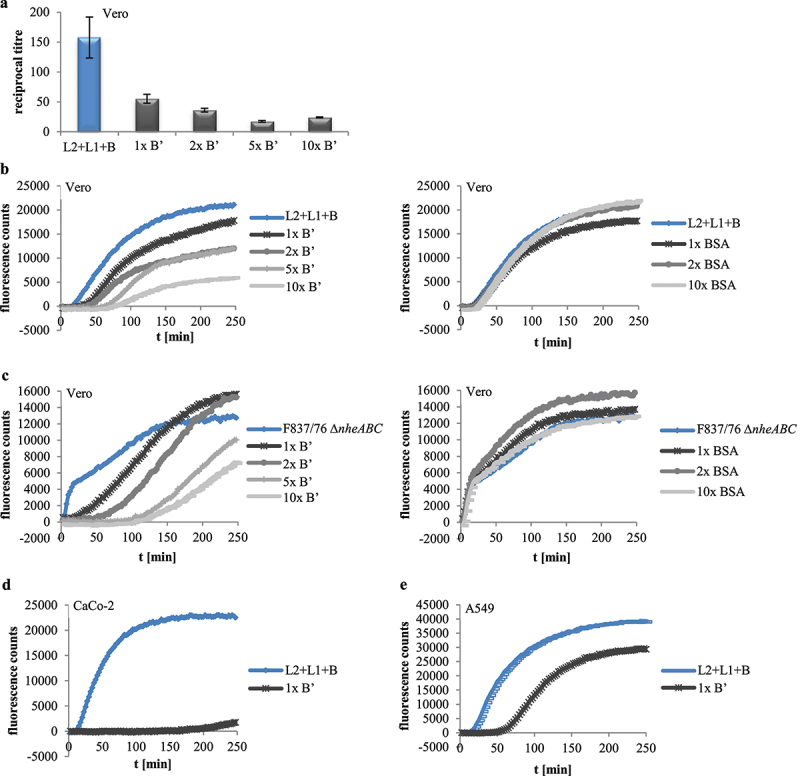
Effects of rHbl B’ on the cytotoxic activity of Hbl. (a). Effects on Vero cells as measured by WST-1 bioassays. The rHbl components L2, L1 and B (1.5 pmol/µl each) were mixed in a 1:1:1 ratio. rHbl B’ was added in increasing ratios. The mixture was applied as dilution series to the cells. (b). PI influx test on Vero cells. rHbl L2, L1, B (1:1:1) and increasing ratios of rHbl B’ were pre-mixed and added in 1:40 dilution to the cells. Increasing ratios of BSA were carried along as control. All proteins had an initial concentration of 1.5 pmol/µl. (c). Experimental setup analogous to b. Instead of rHbl, supernatant of *B. cereus* strain F837/76 ∆*nheABC* was used. (d). Experimental setup analogous to b. Instead of Vero, CaCo-2 cells were used. The 1:1:1:1 protein mixture was tested. (e). Experimental setup analogous to b. Instead of Vero, A549 cells were used. The 1:1:1:1 protein mixture was tested.

After showing that rHbl B’ cannot substitute for any of the other rHbl proteins, all four Hbl components were applied simultaneously. Increasing ratios of rHbl B’ from 1x to 10x compared to the other three components led to decreased titers in WST-1 bioassays on Vero cells (Figure 6), suggesting that the presence of rHbl B’ reduces cytotoxic activity of Hbl. Similar observations were made in PI influx tests. Increasing ratios of rHbl B’ of up to 10x significantly delayed PI influx into Vero cells caused by rHbl L2+L1+B (Figure 6) and also by the supernatant of *B. cereus* strain F837/76 ∆*nheABC* (Figure 6). When BSA was used instead of rHbl B’ (see Figure 6), no delay in PI influx was determined, indicating that the inhibitory effect is highly specific to rHbl B.’ An inhibitory effect of rHbl B’ was also seen on CaCo-2 and A549 cells (Figure 6).

It is noteworthy that strain F837/76 (as well as its *nhe* deletion variant) was initially used for the experiments and especially for cloning of *hblB*, as it is well sequenced and annotated. Nevertheless, in the course of this study, it was shown that in this particular strain, the *hblB* gene is indeed transcribed, but the corresponding Hbl B’ protein is not expressed in detectable levels in the supernatant (see above).

**Figure 7.**
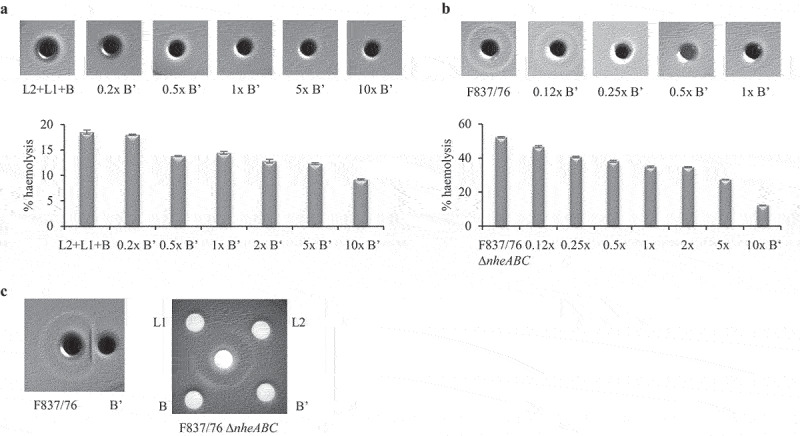
Effect of rHbl B’ on the hemolytic activity of Hbl. (a). rHbl components L2, L1 and B (1.5 pmol/µl each) were mixed in 1:1:1 ratio. 10 µl were applied into stamp holes on sheep blood agar plates together with 0.2x, 0.5x 1x, 5x and 10x rHbl B.’ For quantification, the same setup was used in hemolysis assays with defibrinated sheep blood. Results were compared to the positive control (erythrocytes in H_2_O), which was set to 100%. (b). rHbl B’ (1.5 pmol/µl) was pre-mixed with supernatant of *B. cereus* strains F837/76 or F837/76 ∆*nheABC* in volume ratios from 1:8 to 10:1. Ten µl of the mixtures were applied to the stamp holes. For quantification, samples were applied to hemolysis assays with defibrinated sheep blood. Results were compared to the positive control (erythrocytes in H_2_O), which was set to 100%. (c). Ten µl of rHbl B’ were applied to a stamp hole in approximately 2 mm distance to *B. cereus* F837/76 supernatant. The effect of rHbl B’ was compared to those of rHbl L2, L1 and B on supernatant of *B. cereus* strain F837/76 ∆*nheABC*.

rHbl B’ also showed an influence on hemolytic activity of Hbl on sheep blood agar. When a mixture of rHbl L2, L1, and B was applied together with rising amounts of rHbl B,’ the continuous hemolysis around the stamp hole disappeared. In a quantitative assay with defibrinated sheep blood, rHbl L2+L1+B caused approx. 20% hemolysis compared to the positive control, which was diminished under addition of rising amounts or rHbl B’ (Figure 7).

The same observations were made for supernatants of strains F837/76 and F837/76 ∆*nheABC*. While the Hbl-provoked outer hemolysis circle on sheep blood agar disappeared with rising amounts of rHbl B,’ total hemolysis decreased from approx. 50% to 10%(Figure 7). When rHbl B’ was applied into a separate stamp hole with 2–3 mm distance to the supernatant of F837/76, the outer hemolysis circle seemed to be pushed inwards (Figure 7). This was compared to the previously detected effects of the other three rHbl components [12] on supernatant of strain F837/76 ∆*nheABC*. While rHbl L2 had no effect on the outer circle, rHbl L1 erased and rHbl B enlarged it. The circle was again pushed inwards by rHbl B.’

### rHbl B’ inhibits interaction of rHbl L1 and B instead of B binding to the cell surface

**Figure 8.**
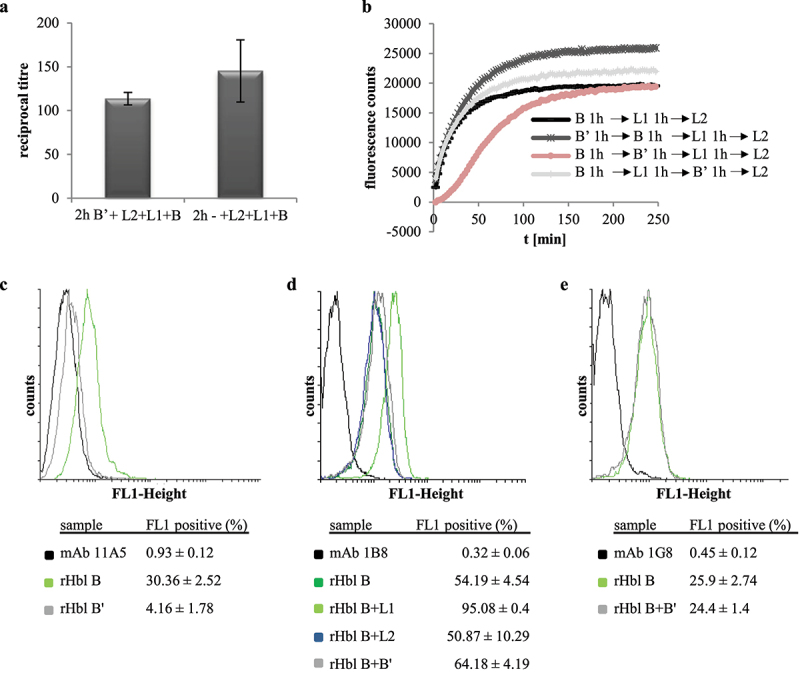
Consecutive and combined application of rHbl B,’ B, L1 and L2 to Vero cells. Concentration of all proteins was 1.5 pmol/µl. (a). WST-1 bioassay. Vero cells were pre-incubated for 2 h with and without 1:40 dilution of rHbl B’ and washed three times in medium before application of a dilution series of rHbl L2+L1+B for 4 h. (b). PI influx test. rHbl components were individually and consecutively applied to the Vero cells in 1:40 dilution each. After each component, cells were washed three times in medium. (c). Flow cytometric analysis of rHbl B and rHbl B’ on Vero cells using mAb 11A5. Black: negative control without antigen. Light green: rHbl B. Grey: rHbl B.’ Mean and standard deviation (fluorescence-positive cells) of two runs, each in duplicates, are shown. (d). Flow cytometric analysis of rHbl on Vero cells using mAb 1B8. Black: negative control without antigen. Dark green: rHbl B. Light green: rHbl B+L1. Blue: rHbl B+L2. Grey: rHbl B+B.’ Means and standard deviations (fluorescence-positive cells) of triplicates are shown. (e). Flow cytometric analysis of rHbl on Vero cells using mAb 1G8. Black: negative control without antigen. Light green: rHbl B. Grey: rHbl B+B.’ Means and standard deviations (fluorescence-positive cells) of triplicates are shown. Additional flow cytometry data can be found in Figure S1. Here, one representative overlay histogram is depicted, respectively.

To gain evidence at which step of Hbl complex/pore formation rHbl B’ might interfere, the rHbl components were applied consecutively in cytotoxicity experiments (seeFigure 8). First, Vero cells were incubated for 2 h with rHbl B’ and then washed in cell culture medium before application of a dilution series of rHbl L2+L1+B for further 4 h and addition of WST-1. The resulting reciprocal titers showed a slight, but non-significant reduction of cytotoxic activity (Figure 8), giving the initial idea that rHbl B’ does not bind to the Vero cells and does not inhibit binding of rHbl B to the cell surface. This was supported by PI influx tests, in which the rHbl components were applied in 1:40 dilution individually and consecutively to the Vero cells. No significant differences were seen when rHbl B’ was not present or applied first or after rHbl L1. On the other hand, a clear delay of PI influx appeared when rHbl B’ was added after B and before L1 (Figure 8). These data further pointed to the conclusion that rHbl B’ influences the interaction of rHbl B and L1 rather than the interaction with L2 or binding of B to the target cell surface. Flow cytometric analyses supported the assumption that rHbl B’ – despite its sequence homology to B – is not able to bind to the target cell surface. Only 4% of Vero cells treated with rHbl B’ were fluorescence-positive, while approximately 30% of cells treated with rHbl B showed fluorescence (Figure 8). In a second approach, mAb 1B8 was used for detection, which is specific to Hbl B, but none of the other Hbl proteins [46]. Thirty-minute incubation with the rHbl proteins resulted in 54% fluorescence-positive cells for rHbl B, and 51% for rHbl B+L2. As observed before [12], addition of rHbl L1 enhanced the binding of B to the cell surface (95%). A slight increase in fluorescence-positive cells was also observed with rHbl B+B’ (64%) (Figure 8). For that reason, a third experiment was conducted using Hbl B-specific mAb 1G8 for detection, which is cross-reactive with L1, but not with B’ [21]. Here, no significant differences were detected between rHbl B and rHbl B+B’ (Figure 8). These experiments allow us to conclude that it is unlikely that rHbl B’ binds to the target cell surface or that it interferes with cell binding of B.

### rHbl B’ directly interacts with rHbl L1

**Figure 9.**
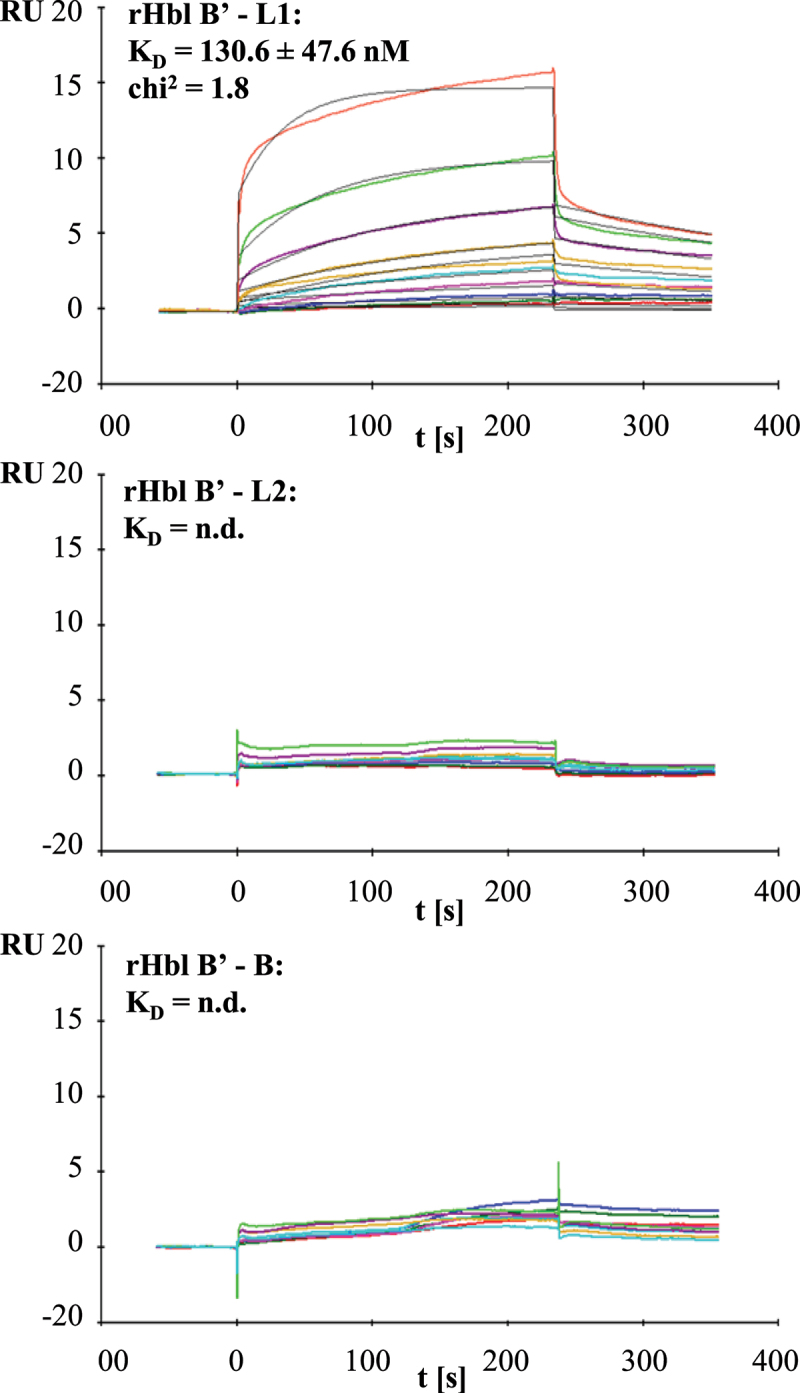
Results of SPR measurements. Binding was tested between rHbl B’ (coupled) and rHbl L1, L2 and B as ligands. Depicted are the representative sensorgrams for pairs B’/L1, B’/L2 and B’/B. or the pair B’/L1 calculated equilibrium dissociation rate (K_D_), error as standard deviation from three independent experiments as well as the chi^2^ value for the curve fit are shown. Concentration series color code: red: 7.8 nM, dark green: 15.6 nM, blue: 31.2 nM, magenta: 62.5 nM, cyan: 125 nM, orange: 250 nM, dark magenta: 500 nM, light green: 1 μM and red: 2 μM. RU: response units.

According to previous results, the interaction of Hbl B and L1 is an important prerequisite for rapid pore formation [12,21]. The above shown data suggested that Hbl B’ hinders this interaction either by binding to Hbl B or L1. Application of rHbl L1-B’ mixtures to enzyme immunoassays and native blotting indicated first evidence for complex formation between Hbl L1 and B’ (data not shown). Surface plasmon resonance (SPR) measurements performed similarly to earlier studies [21] proved this initial observation and verified the binding of rHbl B’ to rHbl L1. Coupling of rHbl B’ to the sensor chip and addition of increasing concentrations of rHbl L1 resulted in a K_D_ value of 130.6 ± 47.6 nM. For rHbl L2 and B, little, rather unspecific interaction was observed, from which K_D_ values could not be calculated (Figure 9).

### Truncated rHbl B’ protein does not exhibit Hbl B function

**Figure 10.**
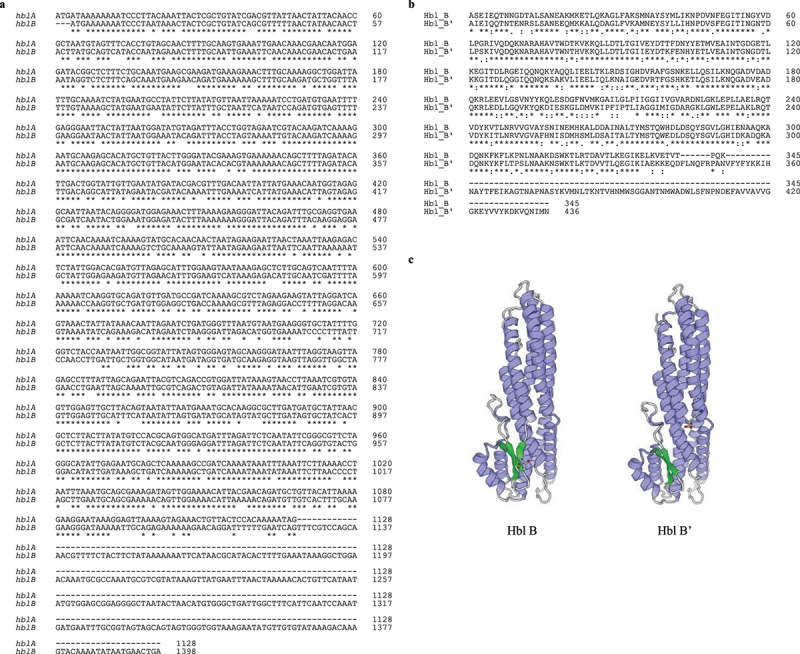
***In silico*** **comparison of Hbl B and Hbl B’ of *B***. **cereus strain F837/76. A**. DNA sequence alignment using clustal omega [46] of *hblA* and the 273 bp longer *hblB* gene. **B.** Amino acid sequence alignment using clustal omega [46] of the corresponding Hbl B and Hbl B’ proteins. **C.** Structural comparison. Protein models were gained using SWISS-MODEL [47]. The N-terminus is in the upper part of each image, the C-terminus is centred in the front of each image, and the C-terminal amino acid is highlighted. Hbl B: Sequence identity to Hbl B with solved crystal structure (PDB ID: 2nrj; [15]) = 99.42 %. Hbl B’: 2nrj was also used as template, sequence identity = 71.39 %. For the C-terminal part, which is not similar to Hbl B (refer to A and B), no template exists and thus, it could not be modeled.

After it became clear that Hbl B’ indeed interferes with pore formation and cytotoxicity of Hbl, the question arose whether this is due to its extended C-terminus presumably developed from duplication of *hblA* and a C-terminal fusion with another ORF [34].Figure 10a shows an alignment of the 1128 bp *hblA* gene and the 273 bp longer *hblB* gene from strain F837/76. In Figure 10b, the corresponding amino acid alignment is depicted. An analysis of the corresponding protein structures revealed high structural similarities between Hbl B and Hbl B.’ It is noteworthy that the C-terminal part, which does not match Hbl B, could not be modeled, as no template exists (Figure 10c).

**Figure 11.**
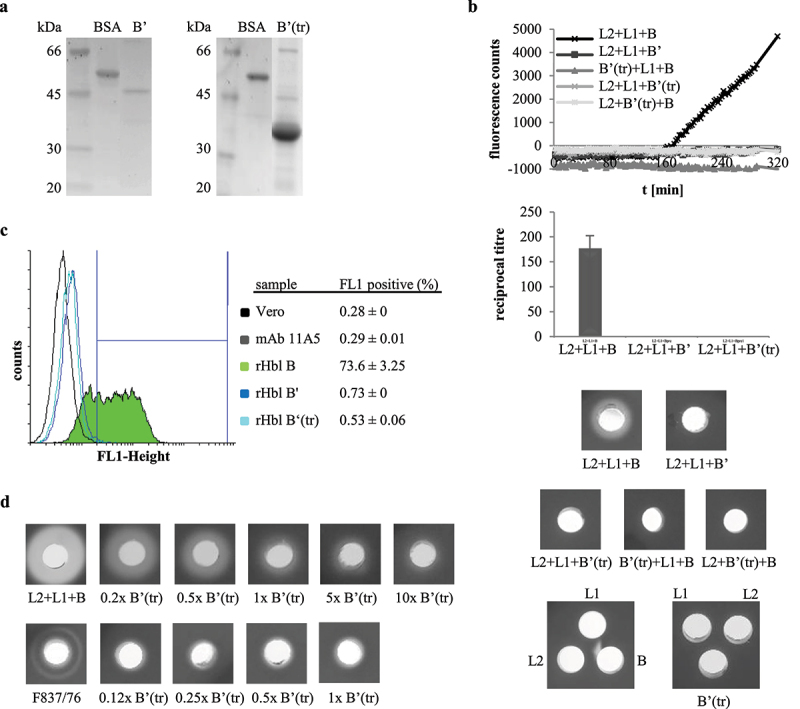
Purification and activity of the truncated protein rHbl B’(tr). Concentration of all proteins was set to 1.5 pmol/μl in each activity test. (a). SDS PAGE and Sypro staining of rHbl B’ (50.6 kDa) and rHbl B’(tr) (41.2 kDa). (b). Replacement of rHbl L2, L1 or B by rHbl B’(tr) on blood agar plates and in WST-1 and PI influx tests on Vero cells. Fluorescence counts are generally lower than in comparable experiments (see Figure 5), as they were obtained in a later approach with less active PI. (c). Flow cytometric analysis of rHbl B’(tr) on Vero cells using mAb 11A5. Black: only Vero cells. Dark gray: negative control without antigen. Light green: rHbl B as control. Fluorescence intensities vary compared to a similar approach (see Figure 8c), as protein incubation times were extended. Blue: rHbl B’ as control. Cyan: rHbl B’(tr). One representative overlay histogram as well as means and standard deviations (fluorescence-positive cells) of triplicates are shown. Additional flow cytometry data can be found in Figure S1. (d). Addition ofrising amounts of rHbl B’(tr) to rHbl or supernatant of strain F837/76 on blood agar plates. Plates with the rHbl samples were photographed after 24 h incubation at 32°C.

To test if that part of Hbl B,’ which equals Hbl B, exhibits a similar function, a truncated version (tr) of rHbl B’ was produced lacking the C-terminal 91 amino acids. Cloning, protein overexpression and purification were performed analogously to the full-length rHbl B’ (Figure 11a). Similarly, rHbl B’(tr) was applied to a selection of the tests described above. Like the full-length rHbl B,’ the truncated version was not able to replace any of the other protein components in the Hbl complex, as shown by PI influx and WST-1 assays and in hemolysis tests on blood agar plates (Figure 11b). Moreover, the protein did not bind to Vero cells (Figure 11c). Again comparably to the full-length protein, the truncated version hindered the hemolytic activity of rHbl or the supernatant of strain F837/76 (Figure 11d). Altogether, despite the similar structure, Hbl B’ seems not to be able to execute Hbl B function, not even when truncated to the same size. This allows us to conclude that not only the additional 91 amino acids C-terminal part, but especially amino acid variations in the N-terminal part of the protein (refer to Figure 10) account for the functional difference.

### Purified Hbl B’ proteins of nine strains repress Hbl activity with varying strength

**Figure 12.**
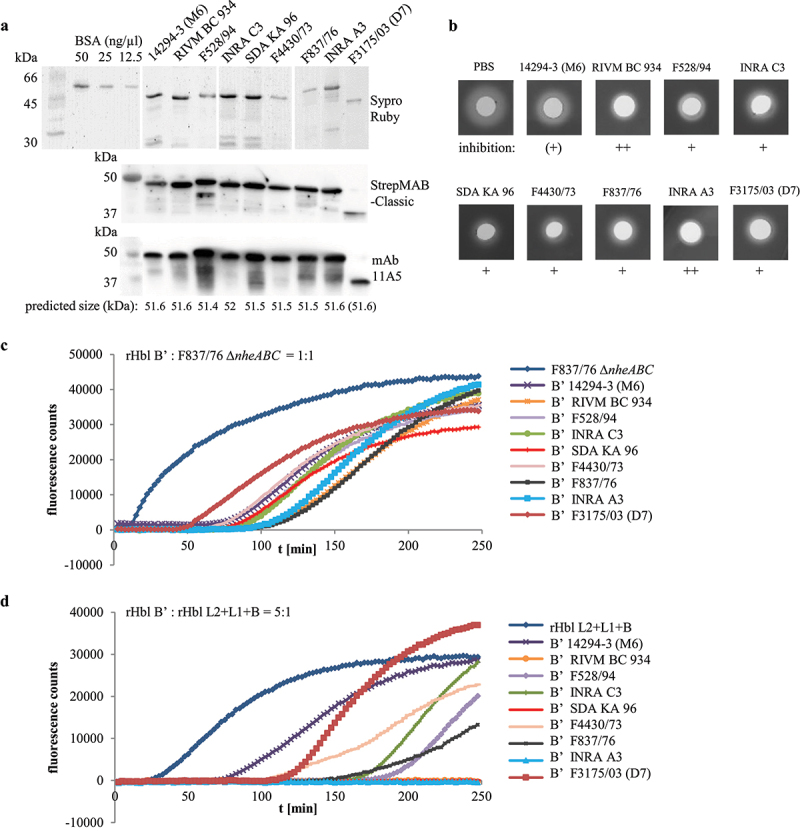
Properties of rHbl proteins from nine *B. cereus* strains. (a). Purified rHbl B’ proteins on SDS PAGE detected by Sypro Ruby, strep-tag specific StrepMAB-Classic and Hbl B/B’- specific mAb 11A5. (b). Inhibition of hemolytic activity by the rHbl B’ proteins. Ten µl of rHbl B’ were filled into the stamp holes on sheep blood agar plates and incubated for 1 h at 32°C before further 10 µl of a mixture of rHbl L2, L1 and B were applied. Plates were photographed after 24 h. Concentration of each recombinant protein: 1.5 pmol/µl. (c). Results of propidium iodide influx tests. Supernatant of strain F837/76 ∆*nheABC* was pre-mixed with rHbl B’ in an 1:1 volume ratio and subsequently applied to Vero cells in 1:40 dilution. (d). Results of propidium iodide influx tests. The rHbl components B, L1 and L2 were pre-mixed with rHbl B’ in a 1:5 ratio and subsequently applied to Vero cells in 1:40 dilution. Concentration of each recombinant protein: 1.5 pmol/µl.

Following the fact that Hbl B’ is produced and secreted in nearly all of the tested *B. cereus* strains, we were interested to see if these proteins exhibit comparable or varying inhibitory activities toward Hbl toxicity. As the *hblB* genes and thus, the Hbl B’ proteins of strains 6/27/S, RIVM BC 934, and RIVM BC 126 are identical (see Figures S2b and c), the Hbl B’ proteins of eight further strains were recombinantly expressed and purified analogously to F837/76 (see above). Figure 12 shows the Hbl B’ proteins stained by Sypro Ruby and detected via StrepMAB-Classic and mAb 11A5 in Western blots. The rHbl B’ proteins of all strains were detected at their predicted size, except strain F3175/03 (D7), for which – despite no stop codon in the DNA sequence – a truncated protein of approx. 37 kDa appeared in all tests (Figure 12a). When the purified rHbl B’ proteins were applied in stamp holes on sheep blood agar plates analogously to rHbl B’ of strain F837/76 (compare Figure 7), they inhibited hemolysis of F837/76 ∆*nheABC* supernatant until a dilution of 1:4, as well as hemolysis of rHbl L2+L1+B (data not shown). Strain-specific differences were first observed when 10 µl rHbl B’ were pre-incubated in the stamp holes for 1 h before application of 10 µl rHbl L2+L1+B. rHbl B’ of strains INRA A3 and RIVM BC 934 strongly inhibited hemolysis, while only a weak effect was observed for rHbl B’ from strain 14294–3 (M6). Inhibition of hemolysis with rHbl B’ of all other strains was interjacent (see Figure 12b). These strain-specific differences in rHbl B’ impact on Hbl pore formation were further investigated in PI influx tests. Generally, PI influx and thus, pore formation was delayed with increasing amounts of rHbl B’ (1x, 5x, and 10x surplus compared to rHbl L2+L1+B or supernatant of strain F837/76 ∆*nheABC* were tested; data not shown). Differences between the rHbl B’ proteins of the nine strains were most obvious at rHbl B:’F837/76 ∆*nheABC* = 1:1 as well as 5x surplus of rHbl B’ compared to rHbl L2+L1+B (see Figure 12c and d). Mixed with the *B. cereus* supernatant, rHbl B’ of strains 14294–3 (M6), F4430/73, and F3175/03 (D7) showed the least inhibition, while pore formation started most tardily under addition of rHbl B’ from strains INRA A3, F837/76, and RIVM BC 934 (Figure 12c). Mixed with rHbl L2+L1+B at 5x surplus, the same three rHbl B’ proteins showed least inhibition (14294–3 (M6), F4430/73, and truncated F3175/03 (D7)), while under addition of rHbl B’ from INRA A3, RIVM BC 934, and SDA KA96 no pore formation could be detected during the 4-h measurement (Figure 12d). For a better overview and comparison of the data, these results are summarized in Table 1.

**Table 1.** Effects of rHbl B’ proteins from nine *B. cereus* strains on Hbl activity. Summary of hemolysis and pore formation experiments depicted in Figure 12. ++: strong inhibitory effect, +: inhibitory effect, (+): weak inhibitory effect.

rHbl B’ from strain	Inhibition of hemolysis (Figure 11)	Inhibition of F837/76 ∆*nheABC* (Figure 11)	Inhibition of rHbl (Figure 11)
14,294–3 (M6)	(+)	(+)	(+)
RIVM BC 934	++	++	++
F528/94	+	+	+
INRA C3	+	+	+
SDA KA 96	+	+	++
F4430/73	+	(+)	(+)
F837/76	+	++	+
INRA A3	++	++	++
F3175/03 (D7)	+	(+)	(+)

Altogether, our data suggest that the fourth component of the *hbl* operon, Hbl B,’ indeed influences Hbl complex and pore formation. Concurrently, significant strain-specific differences occur underlying a yet not understood mechanism. Ongoing research points to different binding capacities toward Hbl L1, which is a very interesting subject for further investigations.

## Discussion

Many years of research have led to a better understanding of the mode of action, especially the mechanism of pore formation, of the enterotoxin complex Hbl from *B. cereus*. It is clear that the three components Hbl B, L1, and L2 bind to the target cell surface in a defined order [9,12]. A defined concentration ratio as well as complex formation between the three single components prior to cell binding also determines the velocity of pore formation and cytotoxic activity [12,20,21]. But can a fourth protein component play an additional role in the mechanism of pore formation? Nearly all bacterial pore-forming toxins are composed of a maximum of two different components [49,50]. Until recently, the *B. cereus* enterotoxins Nhe (non-hemolytic enterotoxin) and Hbl were the only known pore forming toxins consisting of three individual protein components [38,51]. Then, orthologues were detected in Gram-negative bacteria, such as *Serratia marcescens*, *Erwinia mallotivora,* and *Aeromonas hydrophila* after extensive *in silico* search, and the mechanism of the tripartite α-pore forming toxin AhlABC of *A. hydrophila* was characterized [52].

The fourth protein component, Hbl B,’ seems not to be necessary for pore formation and cytotoxic activity of Hbl. This is justified by the occurrence of two distinct *hbl* operons among different strains or also within a single strain, the more common form *hblCDAB* and the rarer variant *hblCDA* missing Hbl B’ [23,29–31]. Moreover, several studies, including our own, proved pore formation and cytotoxicity using the three recombinantly expressed proteins Hbl L2, L1, and B [9,12,20,21]. Nevertheless, the current study showed for the first time that Hbl B’ is indeed expressed by various *B. cereus* strains. One innovation is the development of an easy and functional tool for the detection of Hbl B’ in *B. cereus* culture supernatants. In a previous work, mAbs with specific affinity to Hbl L2, L1, and B were generated [21]. Three of them were able to bind both Hbl B and rHbl B,’ namely, 1K9, 2G10, and 11A5. In the present study, mAb 11A5 proved to be best suited to detect rHbl B’ and also Hbl B’ from culture supernatants. Using this specific mAb in Western blots, two separate bands for Hbl B were detected for strains 14294–3 (M6), 6/27/S, RIVM BC 934, and RIVM BC 126. Whole-genome sequencing and comparison of coverage depths revealed the presence of a second *hbl*_*a*_ operon in strains 14294–3 (M6), 6/27/S, and RIVM BC 126, as well as a very rare, second *nhe*_*a*_ operon in 14294–3 (M6) and MHI 226. Though initially suspected, the *hbl* duplication could not be confirmed in the strain RIVM BC 934 [23,40,42].

With this tool, Hbl B’ could also be detected in the supernatant of 10 *B. cereus* strains, but with varying concentrations. Interestingly, strain F837/76 seemed not to express Hbl B’ in a detectable amount, although *hblB* mRNA existed. In general, concentrations of Hbl B and presumably Hbl B’ in the *B. cereus* supernatants obtained in Western blots in this study are higher than concentrations determined earlier using EIAs and purified proteins as concentration standards. In the latter one, average Hbl B concentrations of approx. .5 ng/µl were obtained (unpublished data). Just recently, we gained evidence that the concentration of Hbl B in *B. cereus* culture supernatants might be drastically underestimated when tested with EIAs, as the protein is highly complex, with itself as well as with Hbl L1 and L2 [20,21].

Furthermore, the present study showed, for the first time, an additional regulatory element in Hbl complex and pore formation via Hbl B'. It is the first one to gain Hbl B’ as single, recombinantly expressed and purified protein. Hbl activity (cytotoxic or hemolytic) was completely abolished when either rHbl B or L2 or L1 was replaced with rHbl B'. Thus, hypotheses were disproved that Hbl B’ might exhibit a similar function than Hbl B [35] due to their sequence homology [34] (see also Figure 10). Instead of sustaining toxicity, our data suggest that Hbl B’ reduces Hbl activity. This was detected in PI influx tests, a method measuring pore-forming activity, as well as in WST-1 bioassays, which show the rate of dead target cells. Hemolytic activity of both rHbl and *B. cereus* culture supernatants was also minimized. To our knowledge, this is the first time that a distinct activity of the *hblB* gene product was observed. Furthermore, we showed that rHbl B’ cannot bind to Vero cells and thus, does not compete with Hbl B for binding sites at the target cell surface. Moreover, it does not seem to influence the binding of B to the cell surface. On the contrary, it rather inhibits the interaction between Hbl B and L1. SPR measurements showed binding of rHbl L1 to rHbl B’ with a K_D_ value of 131 nM (see Figure 9), which is even stronger than binding of rHbl B to L1 observed earlier (K_D_ value of 473 nM [21]), but still relatively low compared to the binding capacity of NheB-C (K_D_ value of 0.48 nM [53]). Thus, the binding of Hbl B’ and L1 seems to be strong enough to affect the interaction between Hbl B and L1.

It has been suggested that *hblB* has originated by duplication of *hblA* and a C-terminal fusion with another ORF [34]. Interactions of Hbl B’ with Hbl L1 might occur because of its sequence homology to Hbl B. The present study further showed that the additional C-terminal 91 amino acids are not (exclusively) responsible for the protein’s inhibitory function (compare Figure 11). We believe that this is rather based on amino acid sequence variations compared to Hbl B (see Figure 10). It has further been suggested that *hblB* has originated last in the evolution of the enterotoxins [23,34] and it has been kept, so it is most likely that an evolutionary advantage exists. According to the data obtained in the present study, it seems to be highly important to instate the Hbl pores precisely via an additional “control mechanism” by Hbl B'. In earlier studies, we showed that Hbl B is present in culture supernatants mostly in large complexes, either with L1 or self-complexed [20]. On the other hand, also a sufficient amount of free Hbl B is necessary for optimal pore formation [12,20], similarly as it has been shown for NheB [54]. Although complexation with Hbl L1 improves binding of Hbl B to the target cell surface, excess of L1 inhibits Hbl activity [12]. The role of the blocking interaction between Hbl B’ and Hbl L1 might thus be to stabilize L1 to partly avoid the formation of huge complexes and to ensure a balance between B-L1 complexes and the free subunits.

In the present study, it was further shown that rHbl B’ proteins from nine *B. cereus* strains decreased the hemolytic and pore-forming activity of Hbl. Only a truncated version of rHbl B’ of strain F3175/03 (D7) could be recombinantly expressed and purified. Since it was detected by StrepMAB-Classic and mAb 11A5 in Western blots (see 12a), it can be concluded that the C-terminal part of the protein is missing. Similarly to, but less actively than the truncated protein rHbl B’(tr), it inhibited pore formation and hemolysis caused by Hbl (12b-d). This allows us to make the assumption that the N-terminal part of Hbl B,’ which is rather similar to Hbl B (refer to Figure 10), is responsible for the interference of B’ with Hbl activity by interacting with Hbl L1. A comparison of the Hbl L1 protein sequences of the different strains is depicted in Figure S2a, which is highly conserved (compare also [42]). On the contrary, Hbl B’ is far less conserved (compare Figure S2b and c). Thus, the genetic basis for the differences in the inhibition of Hbl activity is rather suspected here. Secondly, the high variety of Hbl B’ production might also be found in genetic variations within the intergenic region between *hblA* and *hblB* (compare Figure S2d). Initially, it has been suggested that *hblB* is transcribed together with *hblCDA* as a polycistronic mRNA [28]. Recent studies have found a stem loop resulting in a putative transcriptional stop site upstream of *hblB* from *B. cereus* ATCC 14579 [35] and ATCC 10876 [9]. Clair et al. further identified putative −10 and −35 regions and thus suggested that *hblB* might be transcribed as a single unit [35]. Both of the putative stem loop sequences were identified in the upstream region of *hblB* of our 11 strains. The predicted −10 and −35 regions as well as the predicted transcription start site were identified for nine strains. In general, the length of the *hblB* upstream regions varies and is less conserved than the *hblB* genes (Figure S2d). At this point, it can only be concluded that strain-specific variations in Hbl B’ production and in its inhibitory activity exist, as well as in the sequences of *hblB* and especially the *hbl*A-*hblB* intergenic region. Further in-depth analyses are necessary in order to connect those variations.

Nevertheless, this study is the first one to present a simple tool for the detection of the fourth component of the *hbl* operon, Hbl B,’ in *B. cereus* culture supernatants. We found the protein to be secreted by 10 different strains. More importantly, we could verify an inhibitory function of Hbl B’ in the mechanism of Hbl, which is most likely the balanced orchestration of free Hbl B and Hbl B-L1 complexes in solution.

## Supplementary Material

Supplemental Material

## Data Availability

Data sharing is not applicable to this article as no additional data were created or analyzed in this study.
