# Supplementary material for: Presence and function of Hbl B’, the fourth protein component encoded by the hbl operon in Bacillus cereus
Source: Virulence. 2022 Mar 15;13(1):483–501. doi: 10.1080/21505594.2022.2046951 (PMC8932913; doi:10.1080/21505594.2022.2046951)
Supplement: Supplemental Material [file KVIR_A_2046951_SM6543.zip › Fig._S2_23.02.22.docx]

**A. Hbl L1 protein alignment**

INRA_A3 MKKFPFKVLTLATLATVITATTGNTIHAFAQETTAQEQKVGNYALGPEGLKKALAETGSH 60

INRA_C3 MKKFPFKVLTLATLATVITATTGNTIHAFAQETTAQEQKVGNYALGPEGLKKALAETGSH 60

6/27/S MKKFPFKVLTLATLATVITATTGNTIHAFAQETTAQEQKVGNYALGPEGLKKALAETGSH 60

F3175/03_(D7) MKKFPFKVLTLATLATVITATTGNTIHAFAQETTAQEQKVGNYALGPEGLKKALAETGSH 60

F528/94 MKKFPFKVLTLATLATVITATTGNTIHAFAQETTAQEQKVGNYALGPEGLKKALAETGSH 60

RIVM_BC_934 MKKFPFKVLTLATLATVITATTGNTIHAFAQETTAQEQKVGNYALGPEGLKKALAETGSH 60

SDA_KA_96 MKKFPFKVLTLATLATVITATTGNTIHAFAQETTAQEQKVGNYALGPEGLKKALAETGSH 60

F4430/73 MKKFPFKVLTLATLATVITATTGNTIHAFAQETTAQEQKVGNYALGPEGLKKALAETGSH 60

F837/76 MKKFPFKVLTLATLATVITATTGNTIHAFAQETTAQEQKVGNYALGPEGLKKALAETGSH 60

RIVM_BC_126 MKKFPFKVLTLATLATVITATTGNTIHAFAQETIAQEQKVGNYALGPEGLKKALAETGSH 60

14294-3_(M6) MKKFPFKVLTLATLATVITATTGNTIHAFAQETTAQEQKVGNYALGPEGLKKALAETGSH 60

********************************* **************************

INRA_A3 ILVMDLYAKTMIKQPNVNLSNIDLGSEGGELLKNIHLNQELSRINANYWLDTAKPQIQKT 120

INRA_C3 ILVMDLYAKTMIKQPNVNLSNIDLGSEGGELLKNIHLNQELSRINANYWLDTAKPQIQKT 120

6/27/S ILVMDLYAKTMIKQPNVNLSNIDLGSEGGELLKNIHLNQELSRINANYWLDTAKPQIQKT 120

F3175/03_(D7) ILVMDLYAKTMIKQPNVNLSNIDLGSEGGELLKNIHLNQELSRINANYWLDTAKPQIQKT 120

F528/94 ILVMDLYAKTMIKQPNVNLSNIDLGSEGGELLKNIHLNQELSRINANYWLDTAKPQIQKT 120

RIVM_BC_934 ILVMDLYAKTMIKQPNVNLSNIDLGSEGGELLKNIHLNQELSRINANYWLDTAKPQIQKT 120

SDA_KA_96 ILVMDLYAKTMIKQPNVNLSNIDLGSEGGELLKNIHLNQELSRINANYWLDTAKPQIQKT 120

F4430/73 ILVMDLYAKTMIKQPNVNLSNIDLGSEGGELLKNIHLNQELSRINANYWLDTAKPQIQKT 120

F837/76 ILVMDLYAKTMIKQPNVNLSNINLGSEGGELLKNIHLNQELSRINANYWLDTAKPQIQKT 120

RIVM_BC_126 ILVMDLYAKTMIKQPNVNLSNIDLGSEGGELLKNIHLNQELSRINANYWLDKAKPQIQKT 120

14294-3_(M6) ILVMDLYAKTMIKQPNVNLSNIDLGSEGGELLKNIHLNQELSRINANYWLDKAKPQIQKT 120

**********************:****************************.********

INRA_A3 ARNIVNYDEQFQNYYDTLVETVQKKDKAGLKEGINDLITTINTNSKEVTDVIKMLQDFKG 180

INRA_C3 ARNIVNYDEQFQNYYDTLVETVQKKDKAGLKEGINDLITTINTNSKEVTDVIKMLQDFKG 180

6/27/S ARNIVNYDEQFQNYYDTLVETVQKKDKAGLKEGINDLITTINTNSKEVTDVIKMLQDFKG 180

F3175/03_(D7) ARNIVNYDEQFQNYYDTLVETVQKKDKAGLKEGINDLITTINTNSKEVTDVIKMLQDFKG 180

F528/94 ARNIVNYDEQFQNYYDTLVETVQKKDKAGLKEGINDLITTINTNSKEVTDVIKMLQDFKG 180

RIVM_BC_934 ARNIVNYDEQFQNYYDTLVETVQKKDKAGLKEGINDLITTINTNSKEVTDVIKMLQDFKG 180

SDA_KA_96 ARNIVNYDEQFQNYYDTLVETVQKKDKAGLKEGINDLITTINTNSKEVTDVIKMLQDFKG 180

F4430/73 ARNIVNYDEQFQNYYDTLVETVQKKDKAGLKEGINDLITTINTNSKEVTDVIKMLQDFKG 180

F837/76 ARNIVNYDEQFQNYYDTLVETVQKKDKAGLKEGINDLITTINTNSKEVTDVIKMLQDFKG 180

RIVM_BC_126 ARNIVNYDEQFQNYYDTLVDTVQKKDKAGLKEGINDLITTINTNSKEVTDVIKMLQDFKG 180

14294-3_(M6) ARNIVNYDEQFQNYYDTLVDTVQKKDKAGLKEGINDLITTINTNSKEVTDVIKMLQDFKG 180

*******************:****************************************

INRA_A3 KLYQNSTDFKNNVGGPDGKGGLTAILAGQQATIPQLQAEIEQLRSTQKKHFDDVLAWSIG 240

INRA_C3 KLYQNSTDFKNNVGGPDGKGGLTAILAGQQATIPQLQAEIEQLRSTQKKHFDDVLAWSIG 240

6/27/S KLYQNSTDFKNNVGGPDGKGGLTAILAGQQATIPQLQAEIEQLRSTQKKHFDDVLAWSIG 240

F3175/03_(D7) KLYQNSTDFKNNVGGPDGKGGLTAILAGQQATIPQLQAEIEQLRSTQKKHFDDVLAWSIG 240

F528/94 KLYQNSTDFKNNVGGPDGKGGLTAILAGQQATIPQLQAEIEQLRSTQKKHFDDVLAWSIG 240

RIVM_BC_934 KLYQNSTDFKNNVGGPDGKGGLTAILAGQQATIPQLQAEIEQLRSTQKKHFDDVLAWSIG 240

SDA_KA_96 KLYQNSTDFKNNVGGPDGKGGLTAILAGQQATIPQLQAEIEQLRSTQKKHFDDVLAWSIG 240

F4430/73 KLYQNSTDFKNNVGGPDGKGGLTAILAGQQATIPQLQAEIEQLRSTQKKHFDDVLAWSIG 240

F837/76 KLYQNSTDFKNNVGGPDGKGGLTAILAGQQATIPQLQAEIEQLRATQKKHFDDVLAWSIG 240

RIVM_BC_126 KLYQNSTDFKNNVGGPDGKGGLTAILAGQQATIPQLQAEIEQLRSTQKKHFDDVLAWSIG 240

14294-3_(M6) KLYQNSTDFKNNVGGPDGKGGLTAILAGQQATIPQLQAEIEQLRSTQKKHFDDVLAWSIG 240

********************************************:***************

INRA_A3 GGLGAAILVIAAIGGAVVIVVTGGTATPAVVGGLSALGAAGIGLGTAAGVTASKHMDSYN 300

INRA_C3 GGLGAAILVIAAIGGAVVIVVTGGTATPAVVGGLSALGAAGIGLGTAAGVTASKHMDSYN 300

6/27/S GGLGAAILVIAAIGGAVVIVVTGGTATPAVVGGLSALGAAGIGLGTAAGVTASKHMDSYN 300

F3175/03_(D7) GGLGAAILVIAAIGGAVVIVVTGGTATPAVVGGLSALGAAGIGLGTAAGVTASKHMDSYN 300

F528/94 GGLGAAILVIAAIGGAVVIVVTGGTATPAVVGGLSALGAAGIGLGTAAGVTASKHMDSYN 300

RIVM_BC_934 GGLGAAILVIAAIGGAVVIVVTGGTATPAVVGGLSALGAAGIGLGTAAGVTASKHMDSYN 300

SDA_KA_96 GGLGAAILVIAAIGGAVVIVVTGGTATPAVVGGLSALGAAGIGLGTAAGVTASKHMDSYN 300

F4430/73 GGLGAAILVIAAIGGAVVIVVTGGTATPAVVGGLSALGAAGIGLGTAAGVTASKHMDSYN 300

F837/76 GGLGAAILVIAAIGGAVVIVVTGGTATPAVVGGLSALGAAGIGLGTAAGVTASKHMDSYN 300

RIVM_BC_126 GGLGAAILVIAAIGGAVVIVVTGGTATPAVVGGLSALGAAGIGLGTAAGVTASKHMDSYN 300

14294-3_(M6) GGLGAAILVIAAIGGAVVIVVTGGTATPAVIGGLSALGAAGIGLGTAAGVTASKHMDSYN 300

******************************:*****************************

INRA_A3 EISNKIGELSMKADRANQAVLSLTNAKETLAYLYQTVDQAILSLTNIQKQWNTMGANYTD 360

INRA_C3 EISNKIGELSMKADRANQAVLSLTNAKETLAYLYQTVDQAILSLTNIQKQWNTMGANYTD 360

6/27/S EISNKIGELSMKADRANQAVLSLTNAKETLAYLYQTVDQAILSLTNIQKQWNTMGANYTD 360

F3175/03_(D7) EISNKIGELSMKADRANQAVLSLTNAKETLAYLYQTVDQAILSLTNIQKQWNTMGANYTD 360

F528/94 EISNKIGELSMKADRANQAVLSLTNAKETLAYLYQTVDQAILSLTNIQKQWNTMGANYTD 360

RIVM_BC_934 EISNKIGELSMKADRANQAVLSLTNAKETLAYLYQTVDQAILSLTNIQKQWNTMGANYTD 360

SDA_KA_96 EISNKIGELSMKADRANQAVLSLTNAKETLAYLYQTVDQAILSLTNIQKQWNTMGANYTD 360

F4430/73 EISNKIGELSMKADRANQAVLSLTNAKETLAYLYQTVDQAILSLTNIQKQWNTMGANYTD 360

F837/76 EISNKIGELSMKADRANQAVLSLTNAKETLAYLYQTVDQAILSLTNIQKQWNTMGANYTD 360

RIVM_BC_126 EISNKIGELSMKADRANQAVLSLTNAKETLAYLYQTVDQAILSLTNIQKQWNTMGANYTD 360

14294-3_(M6) EISNKIGELSMKADRANQAVLSLTNAKETLAYLYQTVDQAILSLTNIQKQWNTMGANYTD 360

************************************************************

INRA_A3 LLDNIDSMQDHKFSLIPDDLKAAKESWNDIHKDAEFISKDIAFKQE 406

INRA_C3 LLDNIDSMQDHKFSLIPDDLKAAKESWNDIHKDAEFISKDIAFKQE 406

6/27/S LLDNIDSMQDHKFSLIPDDLKAAKESWNDIHKDAEFISKDIAFKQE 406

F3175/03_(D7) LLDNIDSMQDHKFSLIPDDLKAAKESWNDIHKDAEFISKDIAFKQE 406

F528/94 LLDNIDSMQDHKFSLIPDDLKAAKESWNDIHKDAEFISKDIAFKQE 406

RIVM_BC_934 LLDNIDSMQDHKFSLIPDDLKAAKESWNDIHKDAEFISKDIAFKQE 406

SDA_KA_96 LLDNIDSMQDHKFSLIPDDLKAAKESWNDIHKDAEFISKDIAFKQE 406

F4430/73 LLDNIDSMQDHKFSLIPDDLKAAKESWNDIHKDAEFISKDIAFKQE 406

F837/76 LLDNIDSMQDHKFSLIPDDLKAAKESWNDIHKDAEFISKDIAFKQE 406

RIVM_BC_126 LLDNIDSMQDHKFSLIPDDLKAAKESWNDIHKDAEFISKDIAFKQE 406

14294-3_(M6) LLDNIDSMQDHKFSLIPDDLKAAKESWNDIHKDAEFISKDIAFKQE 406

**********************************************

Grey: signal peptide for secretion according to [1]. Cleavage site according to SignalP-5.0 (http://www.cbs.dtu.dk/services/SignalP/) between aa 30 and 31, AFA-QE. Strain F837/76 is highlighted in green. The few amino acid variations are highlighted in red.

**B. *hblB* gene alignment**

14294-3_(M6) ---ATGAAAAAAAAACCTTATAAAATACTGGCTGTATCAGCATTTTTAATTATGACAACT 57

RIVM_BC_126 ---ATGAAAAAAAAATCTTATAAAATACTGGCTGTATCAGCATTTTTAATTATGACAACT 57

F528/94 ATGATGAAAAAAATTCCTAACAAATTACTCGCTGTATCGACGCTATTAACTATTATAACC 60

F837/76 ---ATGAAAAAAATCCCTAATAAACTACTCGCTGTATCAGCGTTTTTAACTATAACAACT 57

INRA_A3 ATGATGAAAAAAATCCCTCATAAACTACTCGCTGTATCAGCGTTTTTAACTATAACAACT 60

6/27/S ATGATGAAAAAAATCCCTCATAAACTACTCGCTGTATCAGCGTTTTTAACTATAACAACT 60

RIVM_BC_934 ATGATGAAAAAAATCCCTCATAAACTACTCGCTGTATCAGCGTTTTTAACTATAACAACT 60

F3175/03_(D7) ATGATGAAAAAAATCCCTCATAAACTACTCGCTGTATCAGCGTTTTTAACTATAACAACT 60

SDA_KA_96 ATGATAAAAAAAATCCCTAATAAACTATTCGCTGTATCAGCATTTTTAACTATAACAACT 60

INRA_C3 ATGATGAAAAAAATCCCTAATAAACTACTCGCTGTATCAGCGTTTATAACTATAACAACT 60

F4430/73 ATGATGAAAAAAATCCCTAATAAACTACTCGCTGTATCAGCGTTTATAACTATAACAACT 60

** ******* ** * *** ** * ******** * * *** *** * ***

14294-3_(M6) ACCTATGCAGTCACACCAGTAGCAACCTTTGCAATTGAAACTGAACAAACGAACACTGGA 117

RIVM_BC_126 ACCTATGCAGTCACACCAGTAGCAACCTTTGCAATTGAAACTGAACAAACGAACACTGGA 117

F528/94 GCAAATGTAGTTTCACCAGTAACAACTTTTGCAATTGAAATTGGACAAACGAACAATGAA 120

F837/76 ACTTATGCAGTCATACCAATAGAAACTTTTGCAATTGAAATTCAACAAACGAACACTGAA 117

INRA_A3 ACTTATGCAGTCATACCACTAGAAACTTTTGCAATTGAAATTGAACAAACGAACACTGAA 120

6/27/S ACTTATGCAGTCATACCACTAGAAACTTTTGCAATTGAAATTGAACAAACGAACAATGAA 120

RIVM_BC_934 ACTTATGCAGTCATACCACTAGAAACTTTTGCAATTGAAATTGAACAAACGAACAATGAA 120

F3175/03_(D7) ACTTATGCAGTCATACCACTAGAAACTTTTGCAATTGAAATTGAACAAACGAACACTGAA 120

SDA_KA_96 ACTTATGCAGTCATACCAATAGAAACTTTTGCAATTGAAATTGAACAAACGAACATTGAA 120

INRA_C3 ACTTATGCAGTCATACCAATAGAAACTTTTGCAATTGAAATTGAACAAACGAACATTGAA 120

F4430/73 ACTTATGCAGTCATACCAATAGAAACTTTTGCAATTGAAATTGAACAAACGAACATTGAA 120

* *** *** **** ** *** ************* * *********** ** *

14294-3_(M6) GATATGTCTCTTTCAGCAAATGAAGAAAAGATGAAAAAAACTGTACAAGATGCTGGGTTA 177

RIVM_BC_126 GATATGTCTCTTTCAGCAAATGAAGAAAAGATGAAAAAAACTGTACAAGATGCTGGGTTA 177

F528/94 AATATATCTCTTTCAGCAAACGAAGAACAGATGAAAAAAGCTTTGCAAGATGCTGGTTTA 180

F837/76 AATAGGTCTCTTTCAGCAAATGAAGAACAGATGAAAAAAGCTTTGCAAGATGCTGGTTTA 177

INRA_A3 AATATGTCTCTTTCAGCAAATGAAGAACAGATGAAAAAAGCTTTGCAAGATGCTGGTTTA 180

6/27/S AATATGTCTCTTTCAGCAAATGAAGAACAGATGAAAAAAGCTTTGCAAGATGCTGGTTTA 180

RIVM_BC_934 AATATGTCTCTTTCAGCAAATGAAGAACAGATGAAAAAAGCTTTGCAAGATGCTGGTTTA 180

F3175/03_(D7) AATATGTCTCTTTCAGCAAATGAAGAACAGATGAAAAAAGCTTTGCAAGATGCTGGTTTA 180

SDA_KA_96 AATATATCTCTTTCAGCAAATGAAGAACAGATGAAAAAAGCTTTGCAAGATGCTGGTTTA 180

INRA_C3 AATATATCTCTTTCAGCAAATGAAGAACAGATGAAAAAAGCTTTGCAAGATGCTGGTTTA 180

F4430/73 AATATATCTCTTTCAGCAAATGAAGAACAGATGAAAAAAGCTTTGCAAGATGCTGGTTTA 180

*** ************** ****** *********** ** * *********** ***

14294-3_(M6) TTTGCAAAAGCTATGAATGAATATTCTTATTTGCTAATTAATAATCCGGATGTGAGTTTT 237

RIVM_BC_126 TTTGCAAAAGCTATGAATGAATATTCTTATTTGCTAATTAATAATCCGGATGTGAGTTTT 237

F528/94 TTTGCAAAAGCTATGAATGAATATTCTTATTTGCTAATTCATAATCCAGATGTGAGTTTT 240

F837/76 TTTGTAAAAGCTATGAATGAATATTCTTATTTGCTAATTCATAATCCAGATGTGAGTTTT 237

INRA_A3 TTTGAAAAAGCTATGAATGAATATTCTTATTTGCTAATTAATAATCCAGATGTGAGTTTT 240

6/27/S TTTGCAAAAGCTATGAATGAATATTCTTATTTGCTAATTAATAATCCAGATGTGAGTTTT 240

RIVM_BC_934 TTTGCAAAAGCTATGAATGAATATTCTTATTTGCTAATTAATAATCCAGATGTGAGTTTT 240

F3175/03_(D7) TTTGCAAAAGCTATGAATGAATATTCTTATTTGCTAATTAATAATCCAGATGTGAGTTTT 240

SDA_KA_96 TTTGCAAAAGCTATGAGTGAATACTCTTATTTGCTAATTCATAATCCAGATGTGAGTTTT 240

INRA_C3 TTTGCAAAAGCTATGAATGAATACTCTTATTTGCTAATTAATAATCCAGATGTGAGTTTT 240

F4430/73 TTTGCAAAAGCTATGAATGAATACTCTTATTTGCTAATTAATAATCCAGATGTGAGTTTT 240

**** *********** ****** *************** ******* ************

14294-3_(M6) GAAGGAATTTCTATTAATGGGTATGCAGATTTACCTAGTAAAATTGTACAGGATCAAAAG 297

RIVM_BC_126 GAAGGGATTTCTATTAATGGATATGCAGATTTACCTAGTAAAATTGTACAGGATCAAAAG 297

F528/94 GAAGGAATAACTATTAATGGAAATGCAGATTTACCTAGTCAAATTGTACAAGATCAAAAG 300

F837/76 GAAGGAATAACTATTAATGGAAATACAGATTTACCTAGTAAAATTGTACAAGATCAAAAG 297

INRA_A3 GAAGGAATAACTATTAATGGAAATGCAGATTTACCTAGTAAAATTGTACAAGATCAAAAG 300

6/27/S GAAGGAATAACTATTAATGGAAATGCAGATTTACCTAGTAAAATTGTACAAGATCAAAAG 300

RIVM_BC_934 GAAGGAATAACTATTAATGGAAATGCAGATTTACCTAGTAAAATTGTACAAGATCAAAAG 300

F3175/03_(D7) GAAGGAATAACTATTAATGGAAATGCAGATTTACCTAGTAAAATTGTACAAGATCAAAAG 300

SDA_KA_96 GAAGGAATAACTATTAATGGAAATGCAGATTTACCTAGTAAAATTGTACAAGATCAAAAG 300

INRA_C3 GAAGGAATAACTATTAATGGAAATGCAGATTTACCTAGTAAAATTGTACAAGATCAAAAG 300

F4430/73 GAAGGAATAACTATTAATGGAAATGCAGATTTACCTAGTAAAATTGTACAAGATCAAAAG 300

***** ** ********** ** ************** ********** *********

14294-3_(M6) AATGCAAGAGCACATGCTGTTATATGGAATACAAAATTAAAAAAACAGCTTTTAGATACA 357

RIVM_BC_126 AATGCAAGAGCACATGCTGTTACATGGAATACAAAAGTAAAAAAACAGCTTTTAGATACA 357

F528/94 AATGCAAGAGCACATGCTGTTACATGGAATACACAAGTAAAAAAACAGCTTTTAGATACA 360

F837/76 AATGCAAGAGCACATGCTGTTACATGGAATACACACGTAAAAAAACAGCTTTTAGATACA 357

INRA_A3 AATGCAAGAGCACATGCTGTTACATGGAATACACAAGTAAAAAAACAGCTTTTTGATACA 360

6/27/S AATGCAAGAGCACATGCTGTTACATGGAATACACAAGTAAAAAAACAGCTTTTTGATACA 360

RIVM_BC_934 AATGCAAGAGCACATGCTGTTACATGGAATACACAAGTAAAAAAACAGCTTTTTGATACA 360

F3175/03_(D7) AATGCAAGAGCACATGCTGTTACATGGAATACACAAGTAAAAAAACAGCTTTTTGATACA 360

SDA_KA_96 AATGCAAGAGCACATGCTGTTACATGGAATACACAAGTAAAAAAACAACTTTTTGATACA 360

INRA_C3 AATGCAAGAGCACATGCTGTTACATGGAATACACAAGTAAAAAAACAACTTTTTGATACA 360

F4430/73 AATGCAAGAGCACATGCTGTTACATGGAATACACAAGTAAAAAAACAACTTTTTGATACA 360

********************** ********** * ********** ***** ******

14294-3_(M6) TTGACCGGCATTATTGAATACGATACAAAGTTTGAAAATTATTATGAAACATTAGTAGAG 417

RIVM_BC_126 TTGACCGGCATTATTGAATACGATACAAAGTTTGAAAATTATTATGAAACATTAGTAGAG 417

F528/94 TTGACAGGCATTATAGAATACGATACAAAATTTGAAAATCATTATGAAACATTAGTAGAG 420

F837/76 TTGACAGGCATTATAGAATACGATACAAAATTTGAAAATCATTATGAAACATTAGTAGAG 417

INRA_A3 TTGACAGGCATTATAGAATACGATACAAAATTTGAAAATTATTATGAAACATTAGTAGAG 420

6/27/S TTGACAGGCATTATAGAATACGATACAAAATTTGAAAATTATTATGAAACATTAGTAGAG 420

RIVM_BC_934 TTGACAGGCATTATAGAATACGATACAAAATTTGAAAATTATTATGAAACATTAGTAGAG 420

F3175/03_(D7) TTGACAGGCATTATAGAATACGATATAAAATTTGAAAATTATTATGAAACATTAGTAGAG 420

SDA_KA_96 TTGACAGGCATTATAGAATACGATACAAAATTTGAAAATCATTATGAAACATTAGTAGAG 420

INRA_C3 TTGACAGGCATTATAGAATACGATACAAAATTTGAAAATCATTATGAAACATTAGTAGAG 420

F4430/73 TTGACAGGCATTATAGAATACGATACAAAATTTGAAAATCATTATGAAACATTAGTAGAG 420

***** ******** ********** *** ********* ********************

14294-3_(M6) GCGATCAATAATGGAAATGGGGATACTTTAAAAAAGGGGATTACTGATTTAAGGGGGGGA 477

RIVM_BC_126 GCGATCAATAATGGAAATGGGGATACTTTAAAAAAGGGGATTACAGATTTAAGGGTGGGG 477

F528/94 GCAATCAATACTGGAAATGGAGATACTTTAAAAAAAGGGATTACAGATTTACAAGGAGGA 480

F837/76 GCGATCAATACTGGAAATGGAGATACTTTAAAAAAAGGGATTACAGATTTACAAGGAGGA 477

INRA_A3 GCGATCAATACTGGAAATGGAGATACTTTAAAAAAAGGGATTACAGATTTACAGGGAGGA 480

6/27/S GCGATCAATACTGGAAATGGAGATACTTTAAAAAAAGGGATTACAGATTTACAGGGAGGA 480

RIVM_BC_934 GCGATCAATACTGGAAATGGAGATACTTTAAAAAAAGGGATTACAGATTTACAGGGAGGA 480

F3175/03_(D7) GCGATCAATACTGGAAATGGAGATACTTTAAAAAAAGGGATTACAGATTTACAGGGAGGA 480

SDA_KA_96 GCGATCAATACTGGAAATGGAGATACTTTAAAAAAAGGGATTAAAGATCTACAGGGAGGA 480

INRA_C3 GCGATCAATACTGGAAATGGAGATACTTTAAAAAAAGGGATTAAAGATCTACAGGGAGGA 480

F4430/73 GCGATCAATACTGGAAATGGAGATACTTTAAAAAAAGGGATTAAAGATCTACAGGGAGGA 480

** ******* ********* ************** ******* *** ** * **

14294-3_(M6) ATTCAACAAAACCAAAAGTCTGCAAAAGCATTAATAGAAGAATTAACTAAATTTAAAAAC 537

RIVM_BC_126 ATTCAACGAAACCAAGAGTCTGCAAAAGCATTAATAGAAGAATTAACTAAATTTAAAAAC 537

F528/94 ATTCAACAAAATCAAAAGTCTGCAAAAGTATTAATAGAAGAATTAATTCAATTAAAAAAT 540

F837/76 ATTCAACAAAATCAAAAGTCTGCAAAAGTATTAATAGAAGAATTAATTCAATTAAAAAAT 537

INRA_A3 ATTCAACAAAATCAAAAGTCTGCAAAAGCATTAAAAGAAGAATTAATTCAATTAAAAAAT 540

6/27/S ATTCAACAAAATCAAAAGTCTGCAAAAGCATTAATAGAAGAATTAATTCAATTAAAAAAT 540

RIVM_BC_934 ATTCAACAAAATCAAAAGTCTGCAAAAGCATTAATAGAAGAATTAATTCAATTAAAAAAT 540

F3175/03_(D7) ATTCAACAAAATCAAAAGTCTGCAAAAGCATTAAAAGAAGAATTAATTCAATTAAAAAAT 540

SDA_KA_96 ATTCAACAAAATCAAAAGTCTGCAAAAGCATTAATAGAAGAATTAATTCAATTAAAAAAT 540

INRA_C3 ATTCAACAAAATCAAAAGTCTGCAAAAGCATTAATAGAAGAATTAATTCAATTAAAAAAT 540

F4430/73 ATTCAACAAAATCAAAAGTCTGCAAAAGCATTAATAGAAGAATTAATTCAATTAAAAAAT 540

******* *** *** ************ ***** *********** * **** *****

14294-3_(M6) GCTATTGGAGAAGATGTTAGAGTATTTGGAAGCCATAAAGAGACCTTGCAATCGATTTTA 597

RIVM_BC_126 GCTATTGGAGAAGATGTTAGAGTATTTGGAAGCCATAAAGAGACCTTGCAATCGATTTTA 597

F528/94 GCTATTGGAGAAGATGTTAGAACATTTGGAAGTCATAAAGAGACATTGCAATCGATTTTA 600

F837/76 GCTATTGGAGAAGATGTTAGAACATTTGGAAGTCATAAAGAGACATTGCAATCGATTTTA 597

INRA_A3 GCTATTGGAGAAGATGTTAGAACATTTGGAAGTCATAAAGAGACATTGCAATCGATTTTA 600

6/27/S GCTATTGGAGAAGATGTTAGAACATTTGGAAGTCATAAAGAGACATTGCAATCGATTTTA 600

RIVM_BC_934 GCTATTGGAGAAGATGTTAGAACATTTGGAAGTCATAAAGAGACATTGCAATCGATTTTA 600

F3175/03_(D7) GCTATTGGAGAAGATGTTAGAACATTTGGAAGTCATAAAGAGACATTGCAATCGATTTTA 600

SDA_KA_96 GCTATTGGAGAAGATGTTAGAACATTTGGAAGTCATAAAGAGACATTGCAATCGATTTTA 600

INRA_C3 GCTATTGGAGAAGATGTTAGAACATTTGGAAGTCATAAAGAGACATTGCAATCGATTTTA 600

F4430/73 GCTATTGGAGAAGATGTTAGAACATTTGGAAGTCATAAAGAGACATTGCAATCGATTTTA 600

********************* ********* *********** ***************

14294-3_(M6) AAAAACCAAGGAGCTGATGTGGAGACTGATCAAAAGCGTTTAGATGAAGTTTTAGGACAA 657

RIVM_BC_126 AAAAACCAAGGAGCTGATGTAGAGACTGATCAAAAGCGTTTAGATGAAGTTTTAGGACAA 657

F528/94 AAAAACCAAGGTGCTGATGTGGAGGCTGACCAAAAGCGTCTAGAGGACCTTTTAGGACAA 660

F837/76 AAAAACCAAGGTGCTGATGTGGAGGCTGACCAAAAGCGTTTAGAGGACCTTTTAGGACAA 657

INRA_A3 AAAAACCAAGGTGCTGATGTGGAGGCTGACCAAAAGCGTCTAGAGGACCTTTTAGGACAA 660

6/27/S AAAAACCAAGGTGCTGATGTGGAGGCTGACCAAAAGCGTCTAGAGGACCTTTTAGGACAA 660

RIVM_BC_934 AAAAACCAAGGTGCTGATGTGGAGGCTGACCAAAAGCGTCTAGAGGACCTTTTAGGACAA 660

F3175/03_(D7) AAAAACCAAGGTGCTGATGTGGAGGCTGACCAAAAGCGTCTAGAGGACCTTTTAGGACAA 660

SDA_KA_96 AAAAACCAAGGTGCTGATGTGGAGGCGGACCAAAAGCGTCTAGAGGACCTTTTAGGACAA 660

INRA_C3 AAAAACCAAGGTGCTGATGTGGAGGCGGACCAAAAGCGTCTAGAGGACCTTTTAGGACAA 660

F4430/73 AAAAACCAAGGTGCTGATGTGGAGGCGGACCAAAAGCGTCTAGAGGACCTTTTAGGACAA 660

*********** ******** *** * ** ********* **** ** ***********

14294-3_(M6) GTAAACTATTATAAGAAATTAGAATCTGATGGATTAATAATGGTGAAAATACCTTTTATC 717

RIVM_BC_126 GTAAACTATTATAAGAAATTAGAATCTGATGGGTTAATAATGGTGAAAGTCCCTTTTATC 717

F528/94 GTAAAATATCAGAAAGACATAGAATCTAAGGGATTAGATATGGTGAAAATCCCCTTTATT 720

F837/76 GTAAAATATCAGAAAGACATAGAATCTAAGGGATTAGACATGGTGAAAATCCCCTTTATT 717

INRA_A3 GTAAAATATCAGAAAGACATAGAATCTAAGGGCTTAGACATGGTGAAAATCCCATTTATT 720

6/27/S GTAAAATATCAGAAAGACATAGAATCTAAGGGCTTAGACATGGTGAAAATCCCCTTTATT 720

RIVM_BC_934 GTAAAATATCAGAAAGACATAGAATCTAAGGGCTTAGACATGGTGAAAATCCCCTTTATT 720

F3175/03_(D7) GTAAAATATCAGAAAGACATAGAATCTAAGGGCTTAGACATGGTGAAAATCCCCTTTATT 720

SDA_KA_96 GTAAAATATCAGAAAGACATAGAATCTAAGGGGTTAGACATGGTGAAAATTCCCTTTATT 720

INRA_C3 GTAAAATATCAGAAAGACATAGAATCTAAGGGGTTAGACATGGTGAAAATCCCCTTTATT 720

F4430/73 GTAAAATATCAGAAAGACATAGAATCTAAGGGGTTAGACATGGTGAAAATCCCCTTTATT 720

***** *** * ** * ******** * ** *** ********* * ** *****

14294-3_(M6) CCCACGCTTATTTCTGGTGGCATAATGATAGGTACTGCTAGAGATAATTTAGGTCGATTA 777

RIVM_BC_126 CCCACGCTTATTTCTGGTGGCATAATGATAGGTACTGCTAGAGATAATTTAGGTCGATTA 777

F528/94 CCAACCTTGATTGCTGGTGGCATAATGATAGGTGATGCAAGAGGTAAGTTAGGTTGGCTA 780

F837/76 CCAACCTTGATTGCTGGTGGCATAATGATAGGTGATGCAAGAGGTAAGTTAGGTTGGCTA 777

INRA_A3 CCAACCTTGATTGCTGGTGGCATAATGATAGGTGATGCAAGAGGTAAGTTAGGTTGGCTA 780

6/27/S CCAACCTTGATTGCTGGTGGCATAATGATAGGTGATGCAAGAGGTAAGTTAGGTTGGCTA 780

RIVM_BC_934 CCAACCTTGATTGCTGGTGGCATAATGATAGGTGATGCAAGAGGTAAGTTAGGTTGGCTA 780

F3175/03_(D7) CCAACCTTGATTGCTGGTGGCATAATGATAGGTGATGCAAGAGGTAAGTTAGGTTGGCTA 780

SDA_KA_96 CCAACCTTGATTGCTGGTGGCATAATGATAGGTGATGCAAGAGGTAAGTTAGGTTGGCTA 780

INRA_C3 CCAACCTTGATTGCTGGTGGCATAATGATAGGTGATGCAAGAGGTAAGTTAGGTTGGCTA 780

F4430/73 CCAACCTTGATTGCTGGTGGCATAATGATAGGTGATGCAAGAGGTAAGTTAGGTTGGCTA 780

** ** * *** ******************** *** **** *** ****** * **

14294-3_(M6) GAGCCTGCTTTAGCAGAATTACGTAAAACTGTAGATTATAAAATTACATTAAATCGTGTA 837

RIVM_BC_126 GAGCCTGCTTTAGCAGAATTACGTAAAACTGTAGATTATAAAATTACATTAAATCGTGTA 837

F528/94 GAACCTGAATTAGCAAAATTGCGTCAGACTGTAGATTATAAAATAACATTGAATCGTGTA 840

F837/76 GAACCTGAATTAGCAAAATTGCGTCAGACTGTAGATTATAAAATAACATTGAATCGTGTA 837

INRA_A3 GAACCTGAATTAGCAAAATTGCGTCAGACTGTAGATTATAAAATAACATTGAATCGTGTA 840

6/27/S GAACCTGAATTAGAAAAATTGCGTCAGACTGTAGATTATAAAATAACATTGAATCGTGTA 840

RIVM_BC_934 GAACCTGAATTAGAAAAATTGCGTCAGACTGTAGATTATAAAATAACATTGAATCGTGTA 840

F3175/03_(D7) GAACCTGAATTAGAAAAATTGCGTCAGACTGTAGATTATAAAATAACATTGAATCGTGTA 840

SDA_KA_96 GAACCTGAATTAGCAAAATTGCGTCAGACTGTAGATTATAAAATAACATTGAATCGTATA 840

INRA_C3 GAACCTGAATTAGCAAAATTGCGTCAGACTGTAGATTATAAAATAACATTGAATCGTGTA 840

F4430/73 GAACCTGAATTAGCAAAATTGCGTCAGACTGTAGATTATAAAATAACATTGAATCGTGTA 840

** **** **** * **** *** * ***************** ***** ****** **

14294-3_(M6) GTCGGAGTTGCATTTCATAATATTAGTGATATGCATAGTACGATTGATAGTGCTATTACT 897

RIVM_BC_126 GTCGGAGTTGCATTTCATAATATTAGTGATATGCATAGTACGATTGATAGTGCTATTACT 897

F528/94 GTTGGAGTTGCATTTCATAATATTAGTGATATGCATAGTATGCTTGATAGTGCTATCACT 900

F837/76 GTTGGAGTTGCATTTCATAATATTAGTGATATGCATAGTATGCTTGATAGTGCTATCACT 897

INRA_A3 GTTGGAGTTGCATTTCATAATATTAGTGATATGCATAGTATGCTTGATAGTGCTATCACT 900

6/27/S GTTGGAGTTGCATTTCATAATATTAGTGATATGCATAGTATGCTTGATAGTGCTATCACT 900

RIVM_BC_934 GTTGGAGTTGCATTTCATAATATTAGTGATATGCATAGTATGCTTGATAGTGCTATCACT 900

F3175/03_(D7) GTTGGAGTTGCATTTCATAATATTAGTGATATGCATAGTATGCTTGATAGTGCTATCACT 900

SDA_KA_96 GTTGGAGTTGCATTTCATAATATTAGTGATATGCATAGTATGCTTGATAGTGCTATCACT 900

INRA_C3 GTTGGAGTTGCATTTCATAATATTAGTGATATGCATAGTATGCTTGATAGTGCTATCACT 900

F4430/73 GTTGGAGTTGCATTTCATAATATTAGTGATATGCATAGTATGCTTGATAGTGCTATCACT 900

** ************************************* * ************* ***

14294-3_(M6) GCTCTTACTTATATGTCCACACAATGGGATGATTTAGACTCTCAATATTCGGGTGTACTG 957

RIVM_BC_126 GCTCTTACTTATATGTCCACACAATGGGATGATTTAGACTCTCAATATTCGGGCGTACTG 957

F528/94 GCTCTTACTTATATGTCTACGCAATGGGAGGATTTAGATTCTCAATATTCGGGTGTACTG 960

F837/76 GCTCTTACTTATATGTCTACGCAATGGGAGGATTTAGATTCTCAATATTCAGGTGTACTG 957

INRA_A3 GCTCTTACTTATATGTCTACGCAATGGGAGGATTTAGATTCTCAATATTCGGGTGTACTG 960

6/27/S GCTCTTACTTATATGTCTACGCAATGGGAGGATTTAGATTCTCAATATTCGGGTGTACTG 960

RIVM_BC_934 GCTCTTACTTATATGTCTACGCAATGGGAGGATTTAGATTCTCAATATTCGGGTGTACTG 960

F3175/03_(D7) GCTCTTACTTATATGTCTACGCAATGGGAGGATTTAGATTCTCAATATTCGGGTGTACTG 960

SDA_KA_96 GCTCTTACTTATATGTCTACGCAATGGGAGGATTTAGATTCTCAATATTCGGGTGTACTG 960

INRA_C3 GCTCTTACTTATATGTCTACGCAATGGGAGGATTTAGATTCTCAATATTTGGGTGTACTG 960

F4430/73 GCTCTTACTTATATGTCTACGCAATGGGAGGATTTAGATTCTCAATATTCGGGTGTACTG 960

***************** ** ******** ******** ********** ** ******

14294-3_(M6) GGGCATATTGATAAAGCAGATGAAAA------------AGCTGATCAAAATAGATATAAA 1005

RIVM_BC_126 GGGCATATTGATAAAGCTGATGAAAA------------AGCTGATCAAAATAGATATAAA 1005

F528/94 GGACATATTGATAAAGCTGATCAAA------------AGGCTGATCAAAATAAATATAAA 1008

F837/76 GGACATATTGATAAAGCTGATCAAA------------AAGCTGATCAAAATAAATATAAA 1005

INRA_A3 GGACATATTGATAAAGCTGATCAAA------------AAGCTGATCAAAATAAATATAAA 1008

6/27/S GGACATATTGATAAAGCTGATCAAA------------AAGCTGATCAAAATAAATATAAA 1008

RIVM_BC_934 GGACATATTGATAAAGCTGATCAAA------------AAGCTGATCAAAATAAATATAAA 1008

F3175/03_(D7) GGACATATTGATAAAGCTGATCAAA------------AAGCTGATCAAAATAAATATAAA 1008

SDA_KA_96 GGACAAATTGATAAAGCTGATCAAA------------AAGCTGATCAAAATAAATATAAA 1008

INRA_C3 GGACATATTGATAAAGCTGATCAAAAAGCTGATCAAAAAGCTGATCAAAATAAATATAAA 1020

F4430/73 GGACATATTGATAAAGCTGATCAAAA------------AGCTGATCAAAATAAATATAAA 1008

** ** *********** *** *** ************* *******

14294-3_(M6) TTTTTAAAGCCTAACTTGAATTCAGCTAAAGATAGTTGGAAAATATTAAGAACAGATGTT 1065

RIVM_BC_126 TTTTTAAAGCCTAACTTGAATTCAGCTAAAGACAGTTGGAAAACATTAAGAACAGATGTT 1065

F528/94 TTCTTAACCCCTAGCTTGAATGCAGCGAAAAACAGTTGGAAAACATTAAAAACAGATGTT 1068

F837/76 TTCTTAACCCCTAGCTTGAATGCAGCGAAAAACAGTTGGAAAACATTAAAAACAGATGTT 1065

INRA_A3 TTCTTAACCCCTAGCTTGAATGCAGCGAAAAACAGTTGGAAAACATTAAAAACAGATGTT 1068

6/27/S TTCTTAACCCCTAGCTTGAATGCAGCGAAAAACAGTTGGAAAACATTAAAAACAGATGTT 1068

RIVM_BC_934 TTCTTAACCCCTAGCTTGAATGCAGCGAAAAACAGTTGGAAAACATTAAAAACAGATGTT 1068

F3175/03_(D7) TTCTTAACCCCTAGCTTGAATGCAGCGAAAAACAGTTGGAAAACATTAAAAACAGATGTT 1068

SDA_KA_96 TTCTTAACCCCTAGCTTGAATGCAGCGAAAAACAGTTGGAAAACATTAAAAACAGATGTT 1068

INRA_C3 TTCTTAACCCCTAGCTTGAATGCAGCGAAAAACAGTTGGAAAACATTAAAAACAGATGTT 1080

F4430/73 TTCTTAACCCCTAGCTTGAATGTAGCGAAAAACAGTTGGAAAACATTAAAAACAGATGTT 1068

** **** **** ******* *** *** * ********** ***** **********

14294-3_(M6) GTCACATTACTAGAAGGCATAAAAATTGCAGAGAAGAAAGAACAAGATTTTATGAATCTA 1125

RIVM_BC_126 GTCACATTACTAGAAGGCATAAAAATTGCAGAGAAGAAAGAACAAGATTTTATGAATCTA 1125

F528/94 GTCACTTTGCAAGAAGGGATAAAAATTGCAGAGAAAAAAGAAGAGGATTTTTTGAATCAG 1128

F837/76 GTCACTTTGCAAGAAGGGATAAAAATTGCAGAGAAAAAAGAACAGGATTTTTTGAATCAG 1125

INRA_A3 GTCACTTTGCAAGAAGGGATAAAAATTGCAGAGAAAAAAGAACAGGATTTTTTGAATCAG 1128

6/27/S GTCACTTTGCAAGAAGGGATAAAAATTGCAGAGAAAAAAGAACAGGATTTTTTGAATCAG 1128

RIVM_BC_934 GTCACTTTGCAAGAAGGGATAAAAATTGCAGAGAAAAAAGAACAGGATTTTTTGAATCAG 1128

F3175/03_(D7) GTCACTTTGCAAGAAGGGATAAAAATTGCAGAGAAAAAAGAACAGGATTTTTTGAATCAG 1128

SDA_KA_96 GTCACTTTGCAAGAAGGGATAAAAATTGCAGAGAAAAAAGAACAGGATTTTTTGAATCAG 1128

INRA_C3 GTCACTTTGCAAGAAGGGATAAAAATTGCAGAGAAAAAAGAACAGGATTTTTTGAATCAG 1140

F4430/73 GTCACTTTGCAAGAAGGGATAAAAATTGCAGAGAAAAAAGAACAGGATTTTTTGAATCAG 1128

***** ** * ****** ***************** ****** * ****** ******

14294-3_(M6) CTTCGTCCATCAAACGTTTTTTACTTTTATAAAAAAATCCATAACGCATACACTTTTGAA 1185

RIVM_BC_126 CTTCGTCCATCAAATGTTTTTTACTTTTATAAAAAAATCCATAACGCATACACTTTTGAA 1185

F528/94 TTTCGTCCAGCAAACGTTTTCTACTTTTATAAAAAAATTCATAACGCATACACTTTTGAA 1188

F837/76 TTTCGTCCAGCAAACGTTTTCTACTTCTATAAAAAAATTCATAACGCATACACTTTTGAA 1185

INRA_A3 CTTCGTCCATCAAACGTTTTCTACTTTTATAAAAAAATTCATAACGCATACACTTTTGAA 1188

6/27/S CTTCGTCCATCAAACGTGTTCTACTTTTATAAAAAAATTCATAACGCATACACTTTTGAA 1188

RIVM_BC_934 CTTCGTCCATCAAACGTGTTCTACTTTTATAAAAAAATTCATAACGCATACACTTTTGAA 1188

F3175/03_(D7) CTTCGTCCATCAAACGTTTTCTACTTTTATAAAAAAATTCATAACGCATACACTTTTGAA 1188

SDA_KA_96 CTTCGTCCATCAAACGTTTTCTACTTTTATAAAAAAATTCATAACGCATACACTTTTGAA 1188

INRA_C3 CTTCGTCCATCAAACGTTTTCTACTTTTATAAAAAAATTCATAACGCATACACTTTTGAA 1200

F4430/73 CTTCGTCCATCAAACGTTTTCTACTTTTATAAAAAAATTCATAACGCATACACTTTTGAA 1188

******** **** ** ** ***** *********** *********************

14294-3_(M6) ATAAAGACTGGAACAAATGCACCAAATGCGTCTTATAAAGTTATGAATTTAACTAAAAAC 1245

RIVM_BC_126 ATAAAGACAGGAACAAATGCACCAAATGCGTCTTATAAAGTTATGAATTTAACTAAAAAC 1245

F528/94 ATAAAGACTGGAACAAATGCGCCAAATGCGTCTTATAAAGTTATGAATTTAACTAAAAAC 1248

F837/76 ATAAAGGCTGGAACAAATGCGCCAAATGCGTCGTATAAAGTTATGAATTTAACTAAAAAC 1245

INRA_A3 ATAAAGACTGGAACAAATGCGCCAAATGCGTCTTATAAAGTTATGAATTTAACGAAAAAC 1248

6/27/S ATAAAGACTGGAACAAATGCGCCAAATGCGTCTTATAAAGTTATGAATTTAACTAAAAAC 1248

RIVM_BC_934 ATAAAGACTGGAACAAATGCGCCAAATGCGTCTTATAAAGTTATGAATTTAACTAAAAAC 1248

F3175/03_(D7) ATAAAGACTGGAACAAATGCGCCAAATGCGTCTTATAAAGTTATGAATTTAACTAAAAAC 1248

SDA_KA_96 ATAAAGACTGGAACTAATGCGCCAAATGCGTCTTATAAAGTTATGAATTTAACTAAAAAC 1248

INRA_C3 ATAAAGACTGGAACAAATGCGCCAAATGCGTCTTATAAAGTTATGAATTTAACGAAAAAC 1260

F4430/73 ATAAAGACTGGAACAAATGCGCCAAATGCGTCTTATAAAGTTATGAATTTAACGAAAAAC 1248

****** * ***** ***** *********** ******************** ******

14294-3_(M6) ACTGTTCATCATATGTGGAGTGGAGGTGCTAATACAAACATGTGGGCTGACTGGCTTTCA 1305

RIVM_BC_126 ACTGTTCATCATATGTGGAGTGGAGGTGCTAATACAAACATGTGGGCTGACTGGCTTTCA 1305

F528/94 ACTGTTCATAATATGTGGAGTGGAGGGGCTAATACTAACATGTGGGCTGATTGGCTTTCA 1308

F837/76 ACTGTTCATAATATGTGGAGCGGAGGGGCTAATACTAACATGTGGGCTGATTGGCTTTCA 1305

INRA_A3 ACTGTTCATAATATGTGGAGTGGAGGGGCTAATACTAACATGTGGGCTGATTGGCTTTCA 1308

6/27/S ACTGTTCATAATATGTGGAGTGGAGGGGCTAATACTAACATGTGGGCTGATTGGCTTTCA 1308

RIVM_BC_934 ACTGTTCATAATATGTGGAGTGGAGGGGCTAATACTAACATGTGGGCTGATTGGCTTTCA 1308

F3175/03_(D7) ACTGTTCATAATATGTGGAGTGGAGGGGCTAATACTAACATGTGGGCTGATTGGCTTTCA 1308

SDA_KA_96 ACTGTTCATAATATGTGGAGTGGAGGGGCTAATACTAACATGTGGGCTGATTGGCTTTCA 1308

INRA_C3 ACTGTTCATAATATGTGGAGTGGAGGGGCTAATACTAACATGTGGGCTGATTGGCTTTCA 1320

F4430/73 ACTGTTCATAATATGTGGAGTGGAGGGGCTAATACTAACATGTGGGCTGATTGGCTTTCA 1308

********* ********** ***** ******** ************** *********

14294-3_(M6) TTCAATCCAAATGATGAATTTGCGGTGGTAGCAGTAGTGGATGGAAAAGAATATGTTGTA 1365

RIVM_BC_126 TTCAATCCAAATGATGAATTTGCGGTGGTAGCAGTAGTGGATGGAAAAGAATATGTTGTA 1365

F528/94 TTCAATCCAAATGATGAATTTGCGGTAGTAGCAGTAGTGGATGGTAAAGAATATGTTGTG 1368

F837/76 TTCAATCCAAATGATGAATTTGCGGTAGTAGCAGTAGTGGGTGGTAAAGAATATGTTGTG 1365

INRA_A3 TTCAATCCAAATGATGAATTTGCGGTAGTAGCAGTAGTGGATGGTAAAGAATATGTTGTG 1368

6/27/S TTCAATCCAAATGATGAATTTGCGGTAGTAGCAGTAGTGGATGGTAAAGAATATGTTGTG 1368

RIVM_BC_934 TTCAATCCAAATGATGAATTTGCGGTAGTAGCAGTAGTGGATGGTAAAGAATATGTTGTG 1368

F3175/03_(D7) TTCAATCCAAATGATGAATTTGCGGTAGTAGCAGTAGTGGATGGTAAAGAATATGTTGTG 1368

SDA_KA_96 TTCAATCCAAATGATGAATTTGCGGTAGTAGCAGTAGTGGATGGTAAAGAATATGTTGTG 1368

INRA_C3 TTCAATCCAAATGATGAATTTGCGGTAGTAGCAGTAGTGGATGGTAAAGAATATGTTGTG 1380

F4430/73 TTCAATCCAAATGATGAATTTGCGGTAGTAGCAGTAGTGGATGGTAAAGAATATGTTGTG 1368

************************** ************* *** **************

14294-3_(M6) TATAAAGACAAAGTAGAAAATATAATGAACTGA 1398

RIVM_BC_126 TATAAAGACAAAGTAGAAAATATAATGAACTGA 1398

F528/94 TATAAAGACAAAGTACAAAATATAATGAACTGA 1401

F837/76 TATAAAGACAAAGTACAAAATATAATGAACTGA 1398

INRA_A3 TATAAAGACAAAGTACAAAATATAATGAACTGA 1401

6/27/S TATAAAGACAAAGTACAAAATATAATGAACTGA 1401

RIVM_BC_934 TATAAAGACAAAGTACAAAATATAATGAACTGA 1401

F3175/03_(D7) TATAAAGACAAAGTACAAAATATAATGAACTGA 1401

SDA_KA_96 TATAAAGACAAAGTACAAAATATAATGAACTGA 1401

INRA_C3 TATAAAGACAAAGTACAAAATATAATGAACTGA 1413

F4430/73 TATAAAGACAAAGTACAAAATATAATGAACTGA 1401

*************** *****************

**C. Hbl B’ protein alignment**

14294-3_(M6) -MKKKPYKILAVSAFLIMTTTYAVTPVATFAIETEQTNTGDMSLSANEEKMKKTVQDAGL 59

F528/94 MMKKIPNKLLAVSTLLTIITANVVSPVTTFAIEIGQTNNENISLSANEEQMKKALQDAGL 60

F837/76 -MKKIPNKLLAVSAFLTITTTYAVIPIETFAIEIQQTNTENRSLSANEEQMKKALQDAGL 59

6/27/S MMKKIPHKLLAVSAFLTITTTYAVIPLETFAIEIEQTNNENMSLSANEEQMKKALQDAGL 60

RIVM_BC_934 MMKKIPHKLLAVSAFLTITTTYAVIPLETFAIEIEQTNNENMSLSANEEQMKKALQDAGL 60

RIVM_BC_126 -MKKIPHKLLAVSAFLTITTTYAVIPLETFAIEIEQTNNENMSLSANEEQMKKALQDAGL 59

INRA_A3 MMKKIPHKLLAVSAFLTITTTYAVIPLETFAIEIEQTNTENMSLSANEEQMKKALQDAGL 60

F3175/03_(D7) MMKKIPHKLLAVSAFLTITTTYAVIPLETFAIEIEQTNTENMSLSANEEQMKKALQDAGL 60

SDA_KA_96 MIKKIPNKLFAVSAFLTITTTYAVIPIETFAIEIEQTNIENISLSANEEQMKKALQDAGL 60

INRA_C3 MMKKIPNKLLAVSAFITITTTYAVIPIETFAIEIEQTNIENISLSANEEQMKKALQDAGL 60

F4430/73 MMKKIPNKLLAVSAFITITTTYAVIPIETFAIEIEQTNIENISLSANEEQMKKALQDAGL 60

:** * *::***::: : *: .* *: ***** *** : *******:***::*****

14294-3_(M6) FAKAMNEYSYLLINNPDVSFEGISINGYADLPSKIVQDQKNARAHAVIWNTKLKKQLLDT 119

F528/94 FAKAMNEYSYLLIHNPDVSFEGITINGNADLPSQIVQDQKNARAHAVTWNTQVKKQLLDT 120

F837/76 FVKAMNEYSYLLIHNPDVSFEGITINGNTDLPSKIVQDQKNARAHAVTWNTHVKKQLLDT 119

6/27/S FAKAMNEYSYLLINNPDVSFEGITINGNADLPSKIVQDQKNARAHAVTWNTQVKKQLFDT 120

RIVM_BC_934 FAKAMNEYSYLLINNPDVSFEGITINGNADLPSKIVQDQKNARAHAVTWNTQVKKQLFDT 120

RIVM_BC_126 FAKAMNEYSYLLINNPDVSFEGITINGNADLPSKIVQDQKNARAHAVTWNTQVKKQLFDT 119

INRA_A3 FEKAMNEYSYLLINNPDVSFEGITINGNADLPSKIVQDQKNARAHAVTWNTQVKKQLFDT 120

F3175/03_(D7) FAKAMNEYSYLLINNPDVSFEGITINGNADLPSKIVQDQKNARAHAVTWNTQVKKQLFDT 120

SDA_KA_96 FAKAMSEYSYLLIHNPDVSFEGITINGNADLPSKIVQDQKNARAHAVTWNTQVKKQLFDT 120

INRA_C3 FAKAMNEYSYLLINNPDVSFEGITINGNADLPSKIVQDQKNARAHAVTWNTQVKKQLFDT 120

F4430/73 FAKAMNEYSYLLINNPDVSFEGITINGNADLPSKIVQDQKNARAHAVTWNTQVKKQLFDT 120

* ***.*******.*********:*** :****:************* ***::****:**

14294-3_(M6) LTGIIEYDTKFENYYETLVEAINNGNGDTLKKGITDLRGGIQQNQKSAKALIEELTKFKN 179

F528/94 LTGIIEYDTKFENHYETLVEAINTGNGDTLKKGITDLQGGIQQNQKSAKVLIEELIQLKN 180

F837/76 LTGIIEYDTKFENHYETLVEAINTGNGDTLKKGITDLQGGIQQNQKSAKVLIEELIQLKN 179

6/27/S LTGIIEYDTKFENYYETLVEAINTGNGDTLKKGITDLQGGIQQNQKSAKALIEELIQLKN 180

RIVM_BC_934 LTGIIEYDTKFENYYETLVEAINTGNGDTLKKGITDLQGGIQQNQKSAKALIEELIQLKN 180

RIVM_BC_126 LTGIIEYDTKFENYYETLVEAINTGNGDTLKKGITDLQGGIQQNQKSAKALIEELIQLKN 179

INRA_A3 LTGIIEYDTKFENYYETLVEAINTGNGDTLKKGITDLQGGIQQNQKSAKALKEELIQLKN 180

F3175/03_(D7) LTGIIEYDIKFENYYETLVEAINTGNGDTLKKGITDLQGGIQQNQKSAKALKEELIQLKN 180

SDA_KA_96 LTGIIEYDTKFENHYETLVEAINTGNGDTLKKGIKDLQGGIQQNQKSAKALIEELIQLKN 180

INRA_C3 LTGIIEYDTKFENHYETLVEAINTGNGDTLKKGIKDLQGGIQQNQKSAKALIEELIQLKN 180

F4430/73 LTGIIEYDTKFENHYETLVEAINTGNGDTLKKGIKDLQGGIQQNQKSAKALIEELIQLKN 180

******** ****:*********.**********.**:***********.* *** ::**

14294-3_(M6) AIGEDVRVFGSHKETLQSILKNQGADVETDQKRLDEVLGQVNYYKKLESDGLIMVKIPFI 239

F528/94 AIGEDVRTFGSHKETLQSILKNQGADVEADQKRLEDLLGQVKYQKDIESKGLDMVKIPFI 240

F837/76 AIGEDVRTFGSHKETLQSILKNQGADVEADQKRLEDLLGQVKYQKDIESKGLDMVKIPFI 239

6/27/S AIGEDVRTFGSHKETLQSILKNQGADVEADQKRLEDLLGQVKYQKDIESKGLDMVKIPFI 240

RIVM_BC_934 AIGEDVRTFGSHKETLQSILKNQGADVEADQKRLEDLLGQVKYQKDIESKGLDMVKIPFI 240

RIVM_BC_126 AIGEDVRTFGSHKETLQSILKNQGADVEADQKRLEDLLGQVKYQKDIESKGLDMVKIPFI 239

INRA_A3 AIGEDVRTFGSHKETLQSILKNQGADVEADQKRLEDLLGQVKYQKDIESKGLDMVKIPFI 240

F3175/03_(D7) AIGEDVRTFGSHKETLQSILKNQGADVEADQKRLEDLLGQVKYQKDIESKGLDMVKIPFI 240

SDA_KA_96 AIGEDVRTFGSHKETLQSILKNQGADVEADQKRLEDLLGQVKYQKDIESKGLDMVKIPFI 240

INRA_C3 AIGEDVRTFGSHKETLQSILKNQGADVEADQKRLEDLLGQVKYQKDIESKGLDMVKIPFI 240

F4430/73 AIGEDVRTFGSHKETLQSILKNQGADVEADQKRLEDLLGQVKYQKDIESKGLDMVKIPFI 240

*******.********************:*****:::****:* *.:**.** *******

14294-3_(M6) PTLISGGIMIGTARDNLGRLEPALAELRKTVDYKITLNRVVGVAFHNISDMHSTIDSAIT 299

F528/94 PTLIAGGIMIGDARGKLGWLEPELAKLRQTVDYKITLNRVVGVAFHNISDMHSMLDSAIT 300

F837/76 PTLIAGGIMIGDARGKLGWLEPELAKLRQTVDYKITLNRVVGVAFHNISDMHSMLDSAIT 299

6/27/S PTLIAGGIMIGDARGKLGWLEPELEKLRQTVDYKITLNRVVGVAFHNISDMHSMLDSAIT 300

RIVM_BC_934 PTLIAGGIMIGDARGKLGWLEPELEKLRQTVDYKITLNRVVGVAFHNISDMHSMLDSAIT 300

RIVM_BC_126 PTLIAGGIMIGDARGKLGWLEPELEKLRQTVDYKITLNRVVGVAFHNISDMHSMLDSAIT 299

INRA_A3 PTLIAGGIMIGDARGKLGWLEPELAKLRQTVDYKITLNRVVGVAFHNISDMHSMLDSAIT 300

F3175/03_(D7) PTLIAGGIMIGDARGKLGWLEPELEKLRQTVDYKITLNRVVGVAFHNISDMHSMLDSAIT 300

SDA_KA_96 PTLIAGGIMIGDARGKLGWLEPELAKLRQTVDYKITLNRIVGVAFHNISDMHSMLDSAIT 300

INRA_C3 PTLIAGGIMIGDARGKLGWLEPELAKLRQTVDYKITLNRVVGVAFHNISDMHSMLDSAIT 300

F4430/73 PTLIAGGIMIGDARGKLGWLEPELAKLRQTVDYKITLNRVVGVAFHNISDMHSMLDSAIT 300

****:****** ** :** *** * :**:**********:************* :*****

14294-3_(M6) ALTYMSTQWDDLDSQYSGVLGHIDKADE----KADQNRYKFLKPNLNSAKDSWKILRTDV 355

F528/94 ALTYMSTQWEDLDSQYSGVLGHIDKADQ----KADQNKYKFLTPSLNAAKNSWKTLKTDV 356

F837/76 ALTYMSTQWEDLDSQYSGVLGHIDKADQ----KADQNKYKFLTPSLNAAKNSWKTLKTDV 355

6/27/S ALTYMSTQWEDLDSQYSGVLGHIDKADQ----KADQNKYKFLTPSLNAAKNSWKTLKTDV 356

RIVM_BC_934 ALTYMSTQWEDLDSQYSGVLGHIDKADQ----KADQNKYKFLTPSLNAAKNSWKTLKTDV 356

RIVM_BC_126 ALTYMSTQWEDLDSQYSGVLGHIDKADQ----KADQNKYKFLTPSLNAAKNSWKTLKTDV 355

INRA_A3 ALTYMSTQWEDLDSQYSGVLGHIDKADQ----KADQNKYKFLTPSLNAAKNSWKTLKTDV 356

F3175/03_(D7) ALTYMSTQWEDLDSQYSGVLGHIDKADQ----KADQNKYKFLTPSLNAAKNSWKTLKTDV 356

SDA_KA_96 ALTYMSTQWEDLDSQYSGVLGQIDKADQ----KADQNKYKFLTPSLNAAKNSWKTLKTDV 356

INRA_C3 ALTYMSTQWEDLDSQYLGVLGHIDKADQKADQKADQNKYKFLTPSLNAAKNSWKTLKTDV 360

F4430/73 ALTYMSTQWEDLDSQYSGVLGHIDKADQ----KADQNKYKFLTPSLNVAKNSWKTLKTDV 356

*********:****** ****:*****: *****:****.*.** **:*** *:***

14294-3_(M6) VTLLEGIKIAEKKEQDFMNLLRPSNVFYFYKKIHNAYTFEIKTGTNAPNASYKVMNLTKN 415

F528/94 VTLQEGIKIAEKKEEDFLNQFRPANVFYFYKKIHNAYTFEIKTGTNAPNASYKVMNLTKN 416

F837/76 VTLQEGIKIAEKKEQDFLNQFRPANVFYFYKKIHNAYTFEIKAGTNAPNASYKVMNLTKN 415

6/27/S VTLQEGIKIAEKKEQDFLNQLRPSNVFYFYKKIHNAYTFEIKTGTNAPNASYKVMNLTKN 416

RIVM_BC_934 VTLQEGIKIAEKKEQDFLNQLRPSNVFYFYKKIHNAYTFEIKTGTNAPNASYKVMNLTKN 416

RIVM_BC_126 VTLQEGIKIAEKKEQDFLNQLRPSNVFYFYKKIHNAYTFEIKTGTNAPNASYKVMNLTKN 415

INRA_A3 VTLQEGIKIAEKKEQDFLNQLRPSNVFYFYKKIHNAYTFEIKTGTNAPNASYKVMNLTKN 416

F3175/03_(D7) VTLQEGIKIAEKKEQDFLNQLRPSNVFYFYKKIHNAYTFEIKTGTNAPNASYKVMNLTKN 416

SDA_KA_96 VTLQEGIKIAEKKEQDFLNQLRPSNVFYFYKKIHNAYTFEIKTGTNAPNASYKVMNLTKN 416

INRA_C3 VTLQEGIKIAEKKEQDFLNQLRPSNVFYFYKKIHNAYTFEIKTGTNAPNASYKVMNLTKN 420

F4430/73 VTLQEGIKIAEKKEQDFLNQLRPSNVFYFYKKIHNAYTFEIKTGTNAPNASYKVMNLTKN 416

*** **********:**:* :**:******************:*****************

14294-3_(M6) TVHHMWSGGANTNMWADWLSFNPNDEFAVVAVVDGKEYVVYKDKVENIMN 465

F528/94 TVHNMWSGGANTNMWADWLSFNPNDEFAVVAVVDGKEYVVYKDKVQNIMN 466

F837/76 TVHNMWSGGANTNMWADWLSFNPNDEFAVVAVVGGKEYVVYKDKVQNIMN 465

6/27/S TVHNMWSGGANTNMWADWLSFNPNDEFAVVAVVDGKEYVVYKDKVQNIMN 466

RIVM_BC_934 TVHNMWSGGANTNMWADWLSFNPNDEFAVVAVVDGKEYVVYKDKVQNIMN 466

RIVM_BC_126 TVHNMWSGGANTNMWADWLSFNPNDEFAVVAVVDGKEYVVYKDKVQNIMN 465

INRA_A3 TVHNMWSGGANTNMWADWLSFNPNDEFAVVAVVDGKEYVVYKDKVQNIMN 466

F3175/03_(D7) TVHNMWSGGANTNMWADWLSFNPNDEFAVVAVVDGKEYVVYKDKVQNIMN 466

SDA_KA_96 TVHNMWSGGANTNMWADWLSFNPNDEFAVVAVVDGKEYVVYKDKVQNIMN 466

INRA_C3 TVHNMWSGGANTNMWADWLSFNPNDEFAVVAVVDGKEYVVYKDKVQNIMN 470

F4430/73 TVHNMWSGGANTNMWADWLSFNPNDEFAVVAVVDGKEYVVYKDKVQNIMN 466

***.***************************** ***********:****

Grey: signal peptide for secretion according to [1] and [2]. Cleavage site according to SignalP-5.0 (http://www.cbs.dtu.dk/services/SignalP/) between aa 30 and 31, TFA-IE.

**D. sequences between *hblA* and *hblB***

SDA_KA_96 AAACTGTTACTCCACAAAAATAGGGAAATATTAATTCTGTTGTAAAGTGAACTAAAACAT 60

INRA_C3 AAACTGTTACTCCACAAAAATAGGGAAATATTAATTCTGTTGTAAAGTGAACTAAAACAT 60

F4430/73 AAACTGTTACTCCACAAAAATAGGGAAATATTAATTCTGTTGTAAAGTGAACTAAAACAT 60

6/27/S AAACTGTTACTCCACAAAAATAGGGAAATATTAATTCTGTTGTAAAGTGAACTAAAACAT 60

RIVM_BC_934 AAACTGTTACTCCACAAAAATAGGGAAATATTAATTCTGTTGTAAAGTGAACTAAAACAT 60

INRA_A3 AAACTGTTACTCCACAAAAATAGGGAAATATTAATTCTGTTGTAAAGTGAACTAAAACAT 60

F3175/03_(D7) AAACTGTTACTCCACAAAAATAGGGAAATATTAATTCTGTTGTAAAGTGAACTAAAACAT 60

F528/94 AAACTGTTACTCCACAAAAATAGGGAAATATTAATTCTGTTGTAAAGTGAACTAAAACAT 60

F837/76 AAACTGTTACTCCACAAAAATAGGGAAATATTAATTCTGTTGTAAAGTGAACTAAAACAT 60

14294-3_(M6) AAACTGTTACTCCACAAAAATAGGGAAATATTAATTCTGTTGCAAAGTGAACCATAACAT 60

RIVM_BC_126 AAACTGTTACTCCACAAAAATAGGGAAATATTTATTCTGTTGCAAAGTGACCCATAACAT 60

******************************** ********* ******* * * *****

SDA_KA_96 AGAAAGTCTATGATTACACTGTTAAACAGAAAAGTGGTAATTAATAAATCCTTATATAAC 120

INRA_C3 AGAAAGTCTATGATTACACTGTTAAACAGAAAAGGAATAATTGATAAATCCTTATATAAC 120

F4430/73 AGAAAGTCTATGATTACACTGTTAAACAGAAAAGTAATAATTGATAAATCCTTATATAAC 120

6/27/S AGAAAGTCTATGATTACACTGTTAAACAGAAAAGTAATAATTAATAAATCCTTATATAAC 120

RIVM_BC_934 AGAAAGTCTATGATTACACTGTTAAACAGAAAAGTAATAATTAATAAATCCTTATATAAC 120

INRA_A3 AGAAAGTCTATGATTACACTGTTAAACAGAAAAGTAATAATTAATAAATCCTTATATAAC 120

F3175/03_(D7) AGAAAGTCTATGATTACACTGTTAAACAGAAAAGTAATAATTAATAAATCCTTATATAAC 120

F528/94 AGAAAGTCTATGATTACACTGTTAAACAGAAAAGTGGTAATTAATAAATCCTTATATAAC 120

F837/76 AGAAAGTCTATGATTACACTGTGAAACAGAAAAGTGATAATTAATAAATCCTTATATAAC 120

14294-3_(M6) AGAGAGTCTATGATTACACTGTTAAACAGTAAAGCGTTAATTAATAAATCCTTATATATT 120

RIVM_BC_126 AGAGAGTCTATGATTACACTGATAAACAGTAAAGCAGTAATTAATAAATCCTTATATATT 120

*** ***************** ****** **** ***** ***************

SDA_KA_96 AGAAAGGCGGAGTCTCATTAAAGACTTCGCCTTTCAATTATATATAAGTATGATTCGAAT 180

INRA_C3 AGAAAGGCGGAGTCTCATTAAAGACTTCGCCTTTCAATTATATATAAGTATGATTCGAAT 180

F4430/73 AGAAAGGCGGAGTCTCATTAAAGACTTCGCCTTTCAATTATATATAAGTATGATTCGAAT 180

6/27/S AGAAAGGCGGAGTCTCATTAAAGACTTCGCCTTTCAATTATATATAAGTATGATTCGAAT 180

RIVM_BC_934 AGAAAGGCGGAGTCTCATTAAAGACTTCGCCTTTCAATTATATATAAGTATGATTCGAAT 180

INRA_A3 AGAAAGGCGGAGTCTCATTAAAGACTTCGCCTTTCAATTATATATAAGTATGATTCGAAT 180

F3175/03_(D7) AGAAAGGCGGAGTCTCATTAAAGACTTCGCCTTTCAATTATATATAAGTATGATTCGAAT 180

F528/94 AGAAAGGCGGAGTCTCATTAAAGACTTCGCCTTTCAATTATATATAAGTATGATTCGAAT 180

F837/76 AGAAAGGCGGAGTCTCATTAAAGACTTCGCCTTTCAATTATATATAAGTATGATTCGAAT 180

14294-3_(M6) AAAAAGGCGGAGTCTCATTAAAGACTTCGCCTTTCACTTATATATAAGTTATATCCAAAT 180

RIVM_BC_126 AAAAAGGCGGAGTCTCATTAAAGACTTCGCCTTTAACTTATATATAAGTTGTATCCAAAT 180

* ******************************** * ************ ** * ***

SDA_KA_96 AAAAAATAAACTT-GTA-GTTTATTTGTTTTTTAAAATTTTCAACAGAATCGGTTAATTT 238

INRA_C3 AAAA-AATAACCTTGTAGTTTAATTGCTTTTTTAAAATTTGCAACAGAATCGGTTAATTT 239

F4430/73 AAAA-AATAACCTTGTAGTTTAATTGCTTTTTTAAAATTTGCAACAGAATCGGTTAATTT 239

6/27/S AAAG-AATAACCTTGTAGTTTAATTGCTTTTTTAAAATTTGCAATAGAATCGGTTAATTT 239

RIVM_BC_934 AAAG-AATAACCTTGTAGTTTAATTGCTTTTTTAAAATTTGCAATAGAATCGGTTAATTT 239

INRA_A3 AAAA-AATAACCTTGTAGTTTAATTGCTTTTTTAAAATTTGCAACAGAATCGGTTAATTT 239

F3175/03_(D7) AAAA-AATAACCTTGTAGTTTAATTGCTTTTTTAAAATTTGCAATAGAATCGGTTAATTT 239

F528/94 AAAAGAATAACCTTGTAGTTTAATTGCTTTTTTAAAATCTGAAACAGAATGAATTAATTT 240

F837/76 AAAAGAATAACCTTGTAGTTTAATTGCTTTTTTTTAATTTGCAACAGAATCGGTTAATTT 240

14294-3_(M6) AAAAAGATAGCCATGTAGTTTACTTGTT-TTAAAAAATTTGTAACAGAATCGATTAATAG 239

RIVM_BC_126 AAAAAGATAGCCATGTAGTTTACTTGTT-TTTAAAAATTTGTAACAGAATCGATCAATAG 239

*** * * *** ** ** * ** *** * ** ***** * ***

SDA_KA_96 GAAGCAGATTATATACATTTTTTTTAATACTTAATGATTTGAAGGCTGCTAAAGAAAGTT 298

INRA_C3 GAAGTAGATTATATACATTTTCTTTAATACTTAATGATTTGAAGGCTGCTAAAGAAAGTT 299

F4430/73 GAAGTAGATTATATACATTTTCTTTAATACTTAATGATTTAAAGGCTGCTAAAGAAAGTT 299

6/27/S GAAGCAGATGATATACATTTTCTTTAATACTTAATGATTTGAAGGCTGCTAAAGAAAGTT 299

RIVM_BC_934 GAAGCAGATGATATACATTTTCTTTAATACTTAATGATTTGAAGGCTGCTAAAGAAAGTT 299

INRA_A3 GAAGCAGATTATATACATTTTCTTTAATACTTTATGATTTGAAGGCTGCTAAAGAAAGTT 299

F3175/03_(D7) GAAGCAGATTATATACATTTTCTTTAATACTTAATGATTTGAAGGCTGCTAAAGAAAGTT 299

F528/94 GGAGCAGATTATATACATTTTCTTTAATACTTAATGATTTGAAGACTGCTAAAGAAAGTT 300

F837/76 GAAGCTGATTATATACATTTTCTTTAATACTTAATGATTTGAAGACTGCTAAAGAAAGTT 300

14294-3_(M6) AGGACAGGTTATATAGATTTGCTTAATAATATTGAATCTATGCAA--------------- 284

RIVM_BC_126 G--------------------ATAAATAATATCGAATCTATGCAA--------------- 264

* * * * *

SDA_KA_96 CAAACGATACTGATAAAAGATGTAGAACTTATTTTGAAAGACCTCGCTTTTTAAGAAGAT 358

INRA_C3 CAAACGATACTGATAAAAGATGTAGAACTCATTTTGAAAGACATCGCTGTTTAATAAGAT 359

F4430/73 CAAACGATACTGATAAAAGATGTAGAACTCATTTTGAAAGACATCGCTGTTTAATAAGAT 359

6/27/S CAAACGATACTGATAAAAGATGTAGAACTCATTTTGAAAGACATCGCTTTTTAATAAGAT 359

RIVM_BC_934 CAAACGATACTGATAAAAGATGTAGAACTCATTTTGAAAGACATCGCTTTTTAATAAGAT 359

INRA_A3 CAAACGATACTGATAAAAGATGTAGAACTCATTTTGAAAGACATCGCTTTTTAATAAGAT 359

F3175/03_(D7) CAAACGATACTGATAAAAGATGTAGAACTCATTTTGAAAGACATCGCTTTTTAATAAGAT 359

F528/94 CAAACGATACTGATAAAAGATGTAGAACTTATTTTGAAAGACATCGCTTTTTAATAAGAT 360

F837/76 CAAACGATACTAATAAAAGATGTAGAACTCATTTTGAAAGACATCGCTTTTTAATAAGAT 360

14294-3_(M6) --------------------------------------CTACATCGTCTTTTAATAAGAA 306

RIVM_BC_126 --------------------------------------CAACATCGTCTTTTAATAAGAA 286

** *** ***** ****

SDA_KA_96 TAGAACTTGAAAACATACCCTATAGAGGAGGAATACGAA--- 397

INRA_C3 TAGAACTTGAAACCATACCTTATATAGGAGGAATACGAA--- 398

F4430/73 TAGAACTTGAAACCATACCTTATATAGGAGGAATACGAA--- 398

6/27/S TAGAACTTGAAACCATACCTTATATAGGAGGAATACAAA--- 398

RIVM_BC_934 TAGAACTTGAAACCATACCTTATATAGGAGGAATACAAA--- 398

INRA_A3 TAGAACTTGAAACCATACCTTATATAGGAGGAATACGAA--- 398

F3175/03_(D7) TAGAACTTGAAACCATACCTTATATAGGAGGAATACGAA--- 398

F528/94 TAGAACTTGAAACCATACCCTATAGAGGAGGAATACGAA--- 399

F837/76 TAGAACTTGAACCCATACCCTATATAGGAGGAATACGAAAAG 402

14294-3_(M6) TAGGACTTGAAATCAAACTCTATACAGGAGGAATAAGAAAAG 348

RIVM_BC_126 TAGGACTTGAAATCATACTCTATACAGGAGGAATAAGAAAAG 328

*** ******* ** ** **** ********** **

Grey: stem loop according to [3]. Underlined: stem loop according to [2]. Red letters: -10 and -35 motifs according to [2]. Green: transcriptional start site of *hblB* according to [2].

**Fig. S2:** Multiple sequence alignments (clustal omega) of the *hbl* region of 11 *B. cereus* strains. Sequences were obtained from total genome sequencing [4, 5]. (**A)** Hbl L1 amino acid sequences. (**B)** *hblB* genes. (**C)** Hbl B’ amino acid sequences. (**D)** Genetic region between *hblA* and *hblB*.

1. Økstad, O.A., et al., *Sequence analysis of three Bacillus cereus loci carrying PIcR-regulated genes encoding degradative enzymes and enterotoxin.* Microbiology, 1999. **145** p. 3129-38.

2. Clair, G., et al., *Expanding the known repertoire of virulence factors produced by Bacillus cereus through early secretome profiling in three redox conditions.* Mol Cell Proteomics, 2010. **9**(7): p. 1486-98.

3. Sastalla, I., et al., *The Bacillus cereus Hbl and Nhe tripartite enterotoxin components assemble sequentially on the surface of target cells and are not interchangeable.* PLoS One, 2013. **8**(10): p. e76955.

4. Böhm, M.E., et al., *Massive horizontal gene transfer, strictly vertical inheritance and ancient duplications differentially shape the evolution of Bacillus cereus enterotoxin operons hbl, cytK and nhe.* BMC Evol Biol, 2015. **15**: p. 246.

5. Jessberger, N., et al., *From genome to toxicity: a combinatory approach highlights the complexity of enterotoxin production in Bacillus cereus.* Front Microbiol, 2015. **6**: p. 560.
