# Supplementary figures and images for: Presence and function of Hbl B’, the fourth protein component encoded by the hbl operon in Bacillus cereus
Source: Virulence. 2022 Mar 15;13(1):483–501. doi: 10.1080/21505594.2022.2046951 (PMC8932913; doi:10.1080/21505594.2022.2046951)

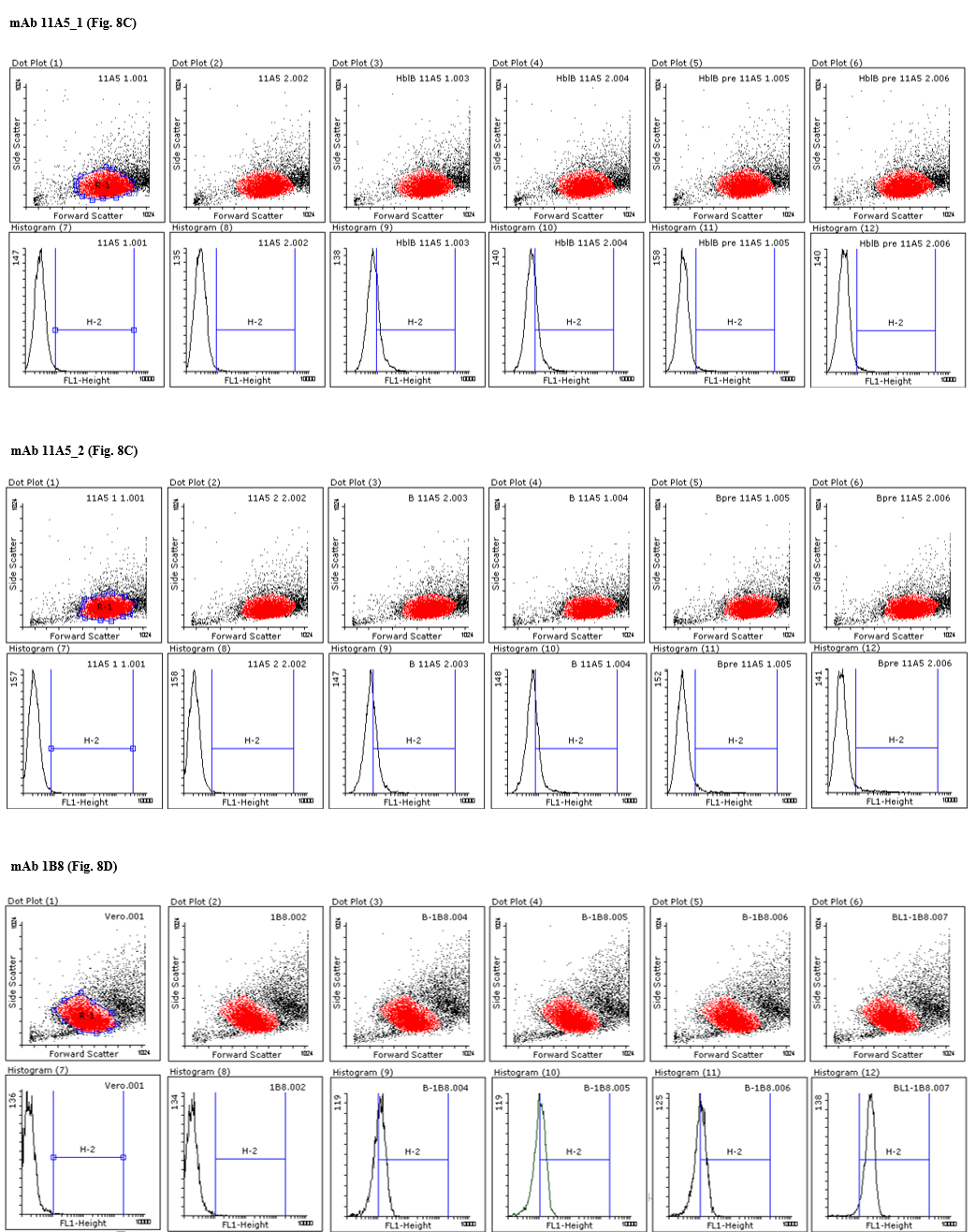

Supplement: Supplemental Material [file KVIR_A_2046951_SM6543.zip › Fig.S1_A.tif]

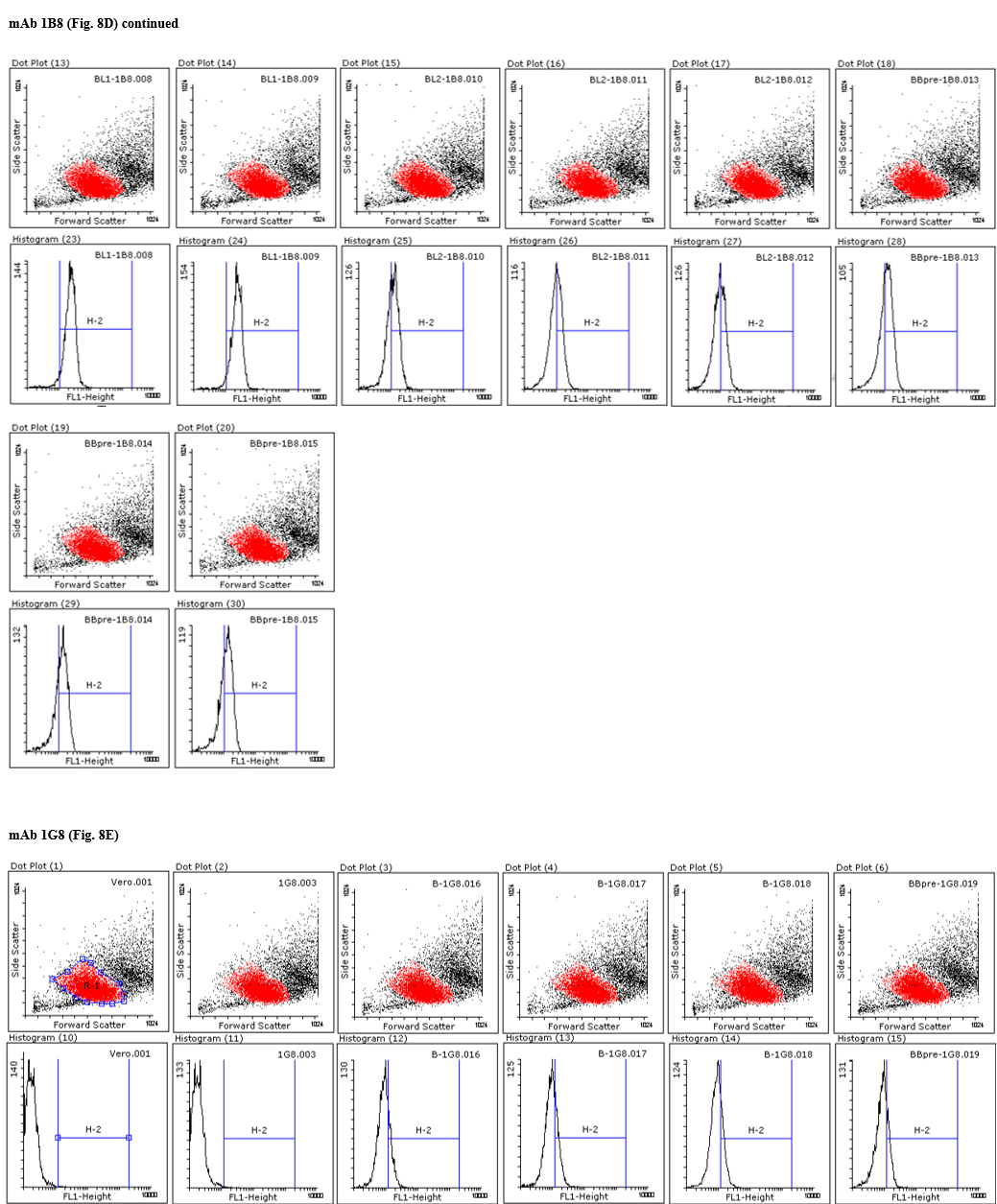

Supplement: Supplemental Material [file KVIR_A_2046951_SM6543.zip › Fig.S1_B.tif]

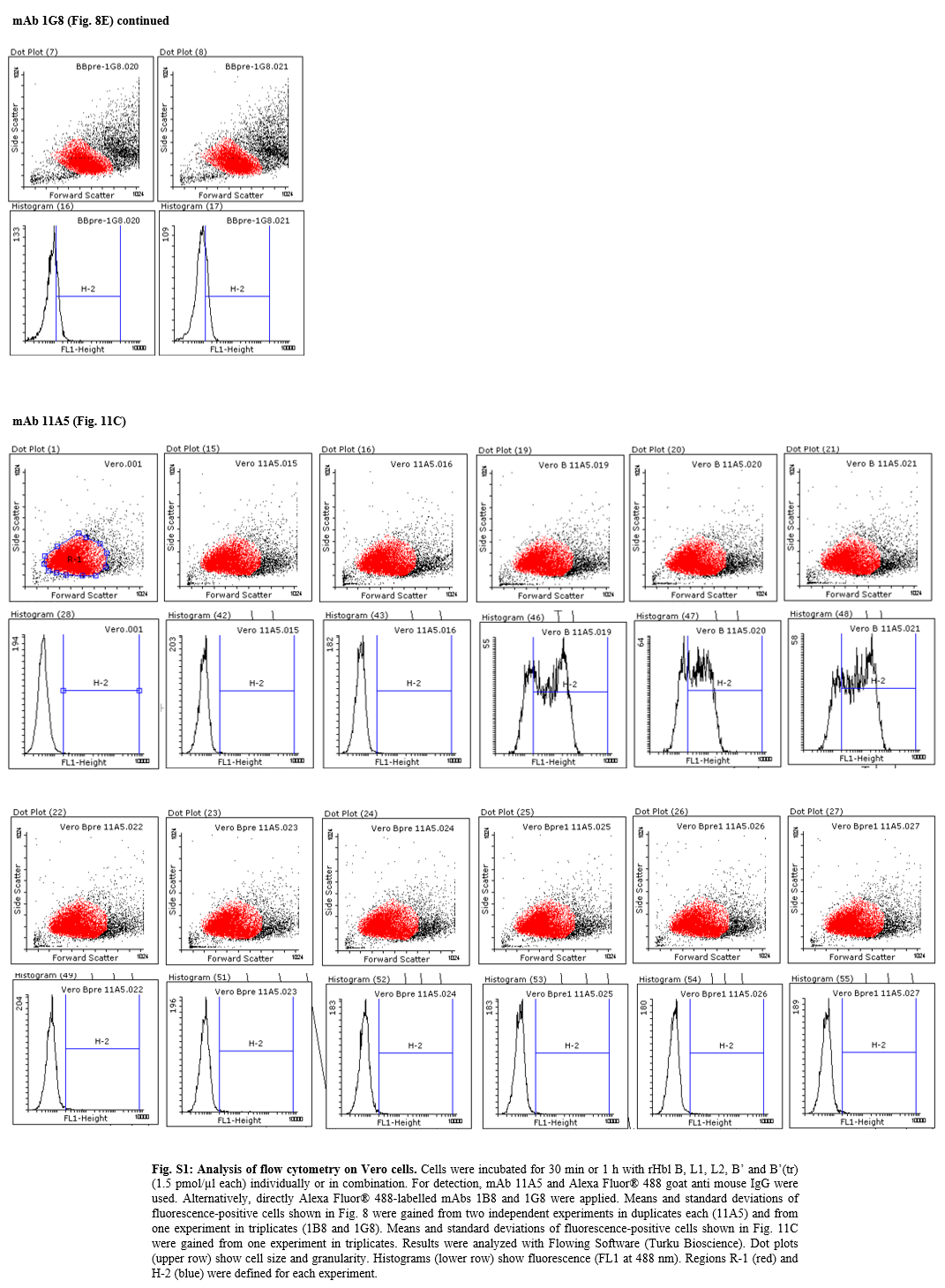

Supplement: Supplemental Material [file KVIR_A_2046951_SM6543.zip › Fig.S1_C.tif]
